# Innovations in skin microphysiological systems for nonclinical testing and FDA modernization

**DOI:** 10.1038/s41378-025-01149-1

**Published:** 2026-01-28

**Authors:** Taeim Lee, Sang Yoon Kyung, Minseo Kwon, Byoungjun Park, Jihoon Ko

**Affiliations:** 1 Department of BioNano Technology, Gachon University, Seongnam-si, Gyeonggi-do 13120 Republic of Korea; 2 Skin & Natural Products Laboratory, Kolmar Korea Co., Ltd., 61, Heolleung-ro 8-gil, Seocho-gu, Seoul, 06800 Republic of Korea

**Keywords:** Microfluidics, Environmental, health and safety issues

## Abstract

Recent innovations in skin microphysiological systems (MPSs) have gained momentum following regulatory advances such as the FDA Modernization Act 2.0 and the global shift toward alternatives to animal testing. This review highlights the development of three major technologies—3D bioprinting, skin organoids, and skin-on-a-chip—and their roles in replicating human skin physiology for research and preclinical applications. We examine how these platforms model complex skin functions, including epidermal barrier formation, vascular and immune interactions, and disease phenotypes such as psoriasis, atopic dermatitis, melanoma, and viral infections. In addition to summarizing their utility in toxicological screening and therapeutic evaluation, we explore how current OECD test guidelines may guide future validation efforts. Finally, we discuss emerging strategies for integrating automation and machine learning-based image analysis to enable scalable, high-content screening of skin MPS models across diverse applications.

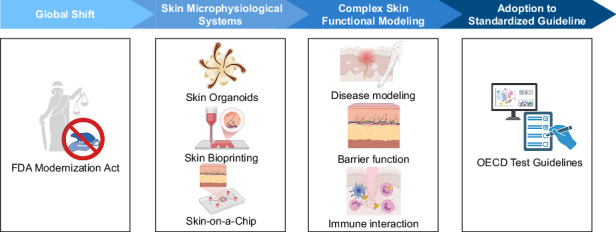

## Introduction

Conventional methods for evaluating skin irritancy and corrosiveness, such as animal testing and Transwell-based human skin equivalents (HSEs), remain widely used but have intrinsic limitations. These models often fail to capture the structural and functional complexity of human skin, including its dynamic responses to long-term stimuli, vascular interactions, and immune involvement. In contrast, microphysiological systems (MPSs) are in vitro models that replicate the three-dimensional (3D) architecture, cellular diversity, and functional responses of human tissues under physiologically relevant conditions. These platforms often incorporate elements such as fluid perfusion, mechanical stimulation, or multicellular co-culture to reproduce tissue-level behaviors. Skin MPS are a subset of MPS that specifically model human skin, including its stratified structure, barrier function, and interactions with vascular, lymphatic, and immune components^1,2^. Skin MPS encompasses three major approaches: skin-on-a-chip devices, bioprinted skin constructs, and skin organoids. By capturing these integrated features, skin MPS provides more physiologically relevant, dynamic, and reproducible platforms than conventional 2D cultures or static Transwell-based HSEs^3,4^.

The enactment of the FDA Modernization Act 2.0 and the Modernization of Cosmetics Regulation Act (MoCRA) represents a pivotal shift in preclinical testing, formally supporting non-animal approaches, including MPS and organotypic models^5,6^. These reforms align with global initiatives such as the EU Cosmetics Regulation and REACH, reflecting an accelerating movement toward ethical and human-relevant testing^7–9^. However, widespread adoption of skin MPS still faces significant real-world barriers. High validation costs, limited inter-laboratory reproducibility, and slow regulatory acceptance hinder their routine use in regulatory submissions. Achieving equivalency or superiority to legacy animal models requires harmonized protocols, standardized readouts, and robust regulatory-grade datasets, which are resource-intensive to generate and maintain. These challenges underscore that while legislative changes support MPS, practical implementation remains an ongoing developmental process.

Amid these challenges, skin MPS are emerging as versatile platforms for mechanistic skin biology studies, long-term toxicology, and disease modeling, with clear potential for regulatory-compliant and industrial applications. This review highlights recent advances in skin-on-a-chip systems, 3D bioprinted skin, and skin organoids, emphasizing their advantages over conventional models and their role in enabling the transition away from animal testing. It also incorporates regulatory and industrial perspectives to provide a realistic framework for translating these advanced in vitro platforms into real-world preclinical and cosmetic testing.

### Current OECD test methods

The OECD guidelines provide a standardized framework for evaluating the preclinical safety of cosmetic and pharmaceutical substances for skin and eye irritation. Key methods include OECD Test Guideline (TG) 430, which measures skin corrosion through transepithelial electrical resistance (TER), and TG 431 and TG 439, which assess skin corrosion and irritation using reconstructed human epidermis (RHE) models. For ocular safety, TG 437 and TG 438 rely on bovine and chicken eye tissues to assess irritation and corrosion, while TG 492 employs reconstructed human cornea-like epithelium (RhCE) models. These tests have successfully established global standards for hazard identification and risk assessment, but their ability to fully capture human tissue complexity is limited.

A major limitation of current OECD test methods is the absence of vascularization and immune system components. Human skin and eyes are highly vascularized and immunologically active tissues. Vascular networks provide nutrient delivery, waste clearance, and systemic signaling, which are essential for tissue repair and recovery after chemical exposure. Immune cells such as macrophages and T cells mediate inflammation, regulate barrier function, and contribute to both acute and chronic responses. Standard RHE or RhCE models in TG 431, TG 439, and TG 492 lack these features, which prevent accurate modeling of inflammation, delayed hypersensitivity, or chronic toxicity. Consequently, compounds that may trigger vascular leakage, prolonged immune activation, or systemic effects can go undetected in these static tests^10–12^.

Ocular models like TG 437 (BCOP) and TG 438 (ICE) rely on non-living animal tissues that cannot regenerate or mount immune responses, and therefore cannot predict cumulative or repeated exposure effects^13^. Similarly, phototoxicity tests like TG 498 capture only short-term damage, overlooking long-term outcomes such as DNA repair, skin aging, or carcinogenesis^14^. Without vascular and immune features, current OECD methods provide only surface-level hazard information, which is insufficient for predicting real-world human responses, especially under repeated or chronic exposure.

These limitations highlight the urgent need to incorporate dynamic, vascularized, and immunocompetent models into regulatory testing^15^. Next-generation skin MPS—including skin-on-a-chip, bioprinted constructs, and skin organoids—offer multi-cellular interactions, perfusion, and immune integration that can overcome the critical gaps of current OECD methods. Table 1 summarizes the key OECD guidelines for skin and ocular safety assessments and the specific physiological gaps that next-generation platforms are designed to address.

**Table 1:** Overview of OECD guidelines for skin and eye irritation testing

OECD guideline	Test method	Application	Limitations/gaps	Refs.
**OECD TG 430**	In vitro skin corrosion: transcutaneous electrical resistance (TER) test	Measures skin corrosion using animal skin	- Simplified skin layers, lacking human complexity. - No immune or vascular interactions. - Cannot simulate healing or dynamic tissue interactions.	^168^
**OECD TG 431**	In vitro skin corrosion: reconstructed human epidermis (RHE) test method	Evaluates skin corrosion with human models	- Focuses on surface damage. - Lacks deep tissue interactions and vascularization. - Cannot model chronic exposure or tissue repair.	^169^
**OECD TG 439**	In vitro skin irritation: reconstructed human epidermis (RHE) test method	Assesses skin irritation	- Limited to 2D and basic 3D models. - No dynamic immune responses. - Lacks long-term effect evaluation.	^170^
**OECD TG 437**	Bovine corneal opacity and permeability (BCOP) test	Measures eye irritation and corrosion	- Uses non-human tissues, reducing relevance to human biology. - No tear film or immune response. - Cannot simulate recovery or dynamic real-time effects.	^171^
**OECD TG 438**	Isolated chicken eye (ICE) test	Evaluates eye corrosion using dead chicken eyes	- Uses non-living, non-human tissues. - No vascular or immune interactions. - Lacks ability to model long-term or dynamic damage.	^172^
**OECD TG 492**	Reconstructed human cornea-like epithelium (RHCE) test method	Tests for eye irritation	- Surface-level evaluation only. - No interactions with deeper eye structures. - Lacks ability to assess long-term effects or recovery.	^173,174^
**OECD TG 498**	Phototoxicity testing in reconstructed human epidermis	Assesses phototoxic effects on skin	- Focuses on short-term effects only. - No modeling of long-term UV exposure or systemic responses. - Lacks ability to measure chronic phototoxicity or damage.	^14^

### Transition to skin MPS in addressing OECD method limitations

The current OECD guidelines provide a structured framework for evaluating skin and ocular irritation, corrosion, and phototoxicity, but they are primarily based on simplified static models and non-human tissues. This results in a limited ability to capture the complex biological interactions that occur in human skin. Key limitations include the inability to model immune cell recruitment, vascular perfusion, tissue repair, and chronic exposure responses. Conventional methods also lack integration with real-time sensing or controlled fluidic flow, which reduces precision and reproducibility.

To meet regulatory requirements within these constraints, preclinical testing has traditionally relied on animal models and HSEs^16,17^. Animal models provide systemic responses but frequently fail to translate to human outcomes, contributing to drug attrition rates of more than 80% in clinical trials for dermatological compounds^18,19^. HSEs, while widely adopted, typically consist of an epidermal layer of keratinocytes over a fibroblast-embedded dermal matrix and can reproduce differentiation markers such as filaggrin and keratic 10. However, they lack vasculature and immune components, making them unable to model inflammation, systemic absorption, or long-term regeneration^20,21^. Furthermore, static Transwell cultures require labor-intensive handling and show batch-to-batch variability, with reported inter-laboratory reproducibility below 70% for irritancy tests^22,23^.

**Fig. 1:**
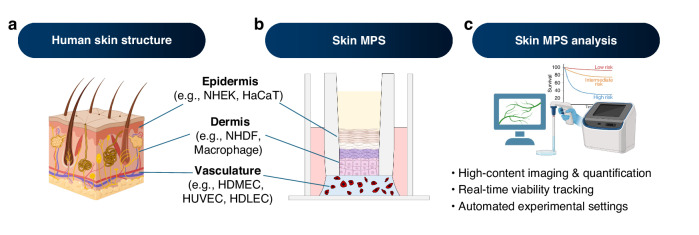
Schematic representation of skin MPS as advanced in vitro test platform. These platforms recapitulate the multilayered architecture of human skin using primary or cell line-derived keratinocytes, fibroblasts, and endothelial cells, and support integrated assay systems for dynamic response tracking. **a** Human skin structure composed of epidermis, dermis, and vasculature, with representative cell types. **b** Skin MPS design mimicking multilayered skin architecture under perfusable conditions. **c** Skin MPS analysis incorporating high-content imaging, real-time viability tracking, and automated readouts. Created with BioRender.com

Advanced MPS overcomes these limitations by recapitulating key structural and dynamic features of native human skin (Fig 1a, b). Skin-on-a-chip platforms incorporate microfluidic perfusion, which supports continuous nutrient delivery and waste removal, and have demonstrated up to a 90% reduction in variability for barrier integrity measurements compared with static HSEs^24,25^. Bioprinted skin allows precise spatial placement of keratinocytes, fibroblasts, melanocytes, and even endothelial cells, achieving layered architecture and enabling high-throughput drug screening with over 85% reproducibility across runs^26^. Skin organoids self-organize from stem or progenitor cells and can capture disease-relevant microanatomy such as basal cell clustering and hair follicle-like structures, allowing chronic toxicity testing that is not possible with current OECD models (Fig. 1c).

**Fig. 2:**
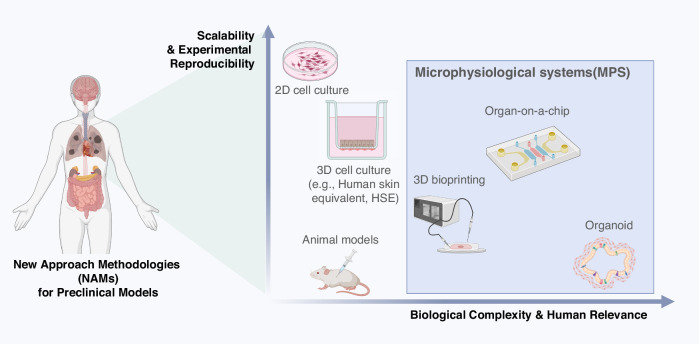
Comparative overview of preclinical models as new approach methods. This illustration compares various preclinical test models in terms of scalability, reproducibility, and physiological relevance to the human body. The models range from traditional approaches such as 2D cell culture and animal testing to more advanced technologies, including 3D cell culture (e.g., human skin equivalent, HSE), 3D bioprinting, organ-on-a-chip systems, and organoids. The diagram highlights a paradigm shift toward MPS that more accurately recapitulates human tissue architecture and function. Created with BioRender.com

By providing human-relevant architecture, dynamic physiological cues, and higher reproducibility, skin MPS enhances predictive accuracy and reduces reliance on animal testing. Figure 2 summarizes how these platforms occupy a unique space with both high physiological relevance and increasing scalability, representing a clear step beyond conventional OECD-guided methods. The following sections detail their core principles, representative applications, and recent innovations.

### 3D Bioprinting technologies for skin research

#### Fundamentals of 3D bioprinting

3D bioprinting is an advanced biofabrication technique that constructs 3D biological tissues by the precise layer-by-layer deposition of bioinks containing living cells and biomaterials^27–29^. This approach enables the spatial organization of multiple cell types and biomaterials to recreate the structural and functional complexity of native tissues^30,31^. In the context of skin research, 3D bioprinting provides a platform to generate constructs that recapitulate the stratified architecture of the epidermis and dermis and, in some cases, incorporate hypodermal components^32^. This capability supports applications in regenerative medicine, pharmaceutical testing, and the development of physiologically relevant in vitro skin models.

#### Bioinks requirements and functional roles in skin bioprinting

Bioinks are the core materials of 3D bioprinting, comprising living cells embedded in biomaterial matrices that provide both structural support and biochemical cues essential for tissue development^33^. For constructing in vitro skin models, bioinks must meet several requirements: (i) biocompatibility to maintain high cell viability and promote proliferation and differentiation, (ii) printability and rheological stability to achieve precise structural fidelity, and (iii) mechanical and degradative properties that support long-term tissue integrity without compromising nutrient diffusion^34^.

**Fig. 3:**
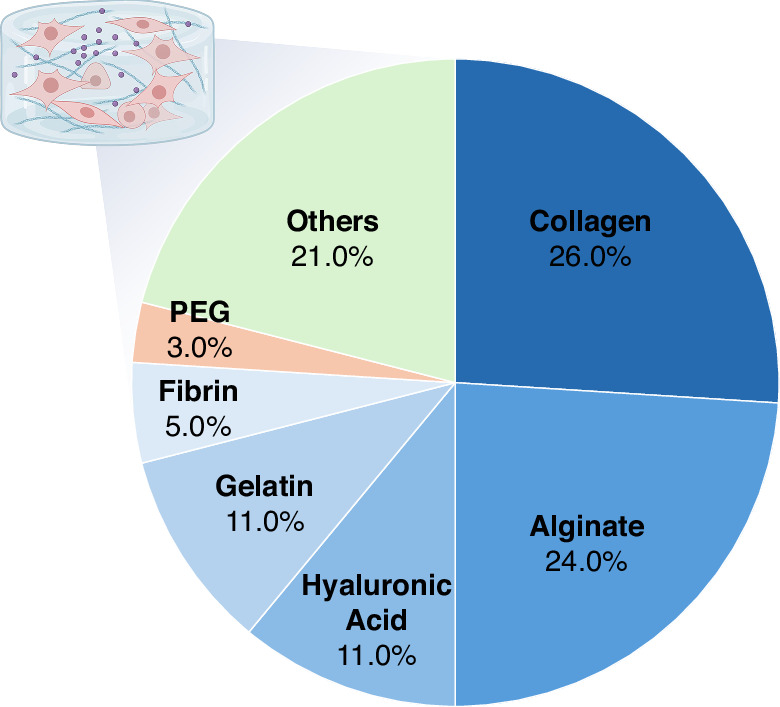
Distribution of commonly used bioink components for skin bioprinting. The pie chart illustrates the proportion use of various biomaterials in skin bioprinting. Collagen and alginate represent the most frequently used components, followed by hyaluronic acid, gelatin, fibrin, polyethylene glycol (PEG), and other materials. These data highlight prevailing trends in bioink formulation for engineered skin tissue models, reflecting preferences based on biocompatibility, printability, and structural support

Each biomaterial contributes uniquely to meeting these requirements. Natural biomaterials such as collagen and alginate dominate skin bioprinting due to their excellent biocompatibility and ability to create hydrogels suitable for keratinocyte and fibroblast encapsulation^35–37^. Collagen promotes cell adhesion and extracellular matrix (ECM) deposition, while alginate allows rapid ionic gelation under mild conditions. Hyaluronic acid (HA) and gelatin are widely employed to enhance hydration, provide bioactive motifs, and support dermal matrix formation^38,39^. Fibrin contributes to wound-healing–related studies by supporting angiogenesis and tissue remodeling^40^, whereas polyethylene glycol (PEG) and its derivatives are used to fine-tune mechanical properties and degradation rates^41^. In clinical settings, acellular dermal matrices such as AlloDerm and Integra serve as gold standards for skin reconstruction and wound healing, and their biomechanical and biochemical characteristics continue to inform the design of next-generation bioinks for regenerative and in vitro applications. A survey of bioink usage in recent skin bioprinting studies is shown in Fig. 3, highlighting collagen (26%) and alginate (24%) as the most prevalent components, followed by hyaluronic acid (11%), gelatin (11%), fibrin (5%), and PEG (3%). The remaining 21% includes other materials such as decellularized ECM and composite hydrogels. These trends underscore the importance of balancing biological fidelity, printability, and structural stability when designing bioinks for engineered skin models.

#### Engineered bioinks for enhanced fidelity

Engineered bioinks are advanced formulations developed to overcome the limitations of single-component bioinks by integrating biological and mechanical functionality^42,43^. These bioinks often combine natural polymers (collagen, gelatin) with synthetic scaffolds (PEG or PEG derivatives) to simultaneously achieve tunable stiffness, controlled degradation, and bioactive signaling^44,45^. Functional enhancements include the incorporation of growth factors, ECM fragments, and peptides, which guide epidermal differentiation, dermal remodeling, and basement membrane formation^46–48^. Rheological optimization, using viscosity modifiers or thixotropic agents, improves extrusion fidelity and prevents cell sedimentation, while tailored crosslinking strategies ensure structural stability.

Physical crosslinking (ionic or thermal) provides rapid yet reversible gel formation^42,49^, chemical crosslinking enhances mechanical robustness, and enzymatic crosslinking using agents such as transglutaminase preserves high cell viability^50,51^. In particular, photo-crosslinkable bioinks such as gelatin methacryloyl (GelMA) and collagen methacrylate (ColMA) have been widely adopted due to their tunable mechanical properties, high printability, and compatibility with UV-based curing^52–55^ By adjusting the degree of methacrylate or UV exposure time, researchers can finely control matrix stiffness to modulate cell-specific behavior. For example, stiffer GelMA substrates promote keratinocyte differentiation and stratification, while softer hydrogels may be preferred for fibroblast migration and ECM remodeling^56,57^. However, crosslinked biomaterials that favor one lineage (e.g., keratinocyte differentiation) may impair fibroblast migration or immune cell infiltration, highlighting the need for balanced tuning of biophysical parameters^58^.

These engineered bioinks directly support functional outcomes that traditional bioinks cannot reliably achieve, including sustained barrier formation, robust dermal–epidermal junction (DEJ) development, and the integration of vascular or immune components. By aligning material properties with biological performance, engineered bioinks are accelerating the fidelity and translational potential of 3D bioprinted skin for applications in drug testing, regenerative medicine, and next-generation in vitro models^59,60^.

#### 3D Bioprinting for HSE

3D bioprinting has significantly advanced the fabrication of human skin equivalents (HSEs) by enabling precise, layer-by-layer deposition of bioinks containing keratinocytes, fibroblasts, and other skin-relevant cell types^61,62^. Unlike standard OECD-defined reconstructed skin models, which typically reproduce the epidermis and dermis in static culture inserts, bioprinted HSEs offer enhanced structural control and the potential for functional complexity. Specifically, bioprinting allows the deliberate recreation of the epidermis, dermis, and in some models the hypodermis, while establishing a well-defined DEJ with basement membrane proteins and interlayer signaling essential for barrier formation^63–65^.

To achieve functional skin construction, bioprinted models meet several key requirements: (i) accurate stratification of keratinocytes and fibroblasts to recapitulate the native epidermal and dermal compartments^66^, (ii) integration of ECM components that support cell adhesion and maturation, (iii) development of a functional DEJ for interlayer communication and basement membrane formation, and (iv) incorporation of elements such as vascular and neural components to maintain nutrient delivery, waste removal, and long-term tissue viability^67–70^. However, the limited ability of current 3D bioprinting techniques to fully replicate the spatial microarchitecture of native skin may restrict cell-cell and cell-matrix interactions, posing a bottleneck in recapitulating multi-cellular communication within skin MPS platforms.

**Fig. 4:**
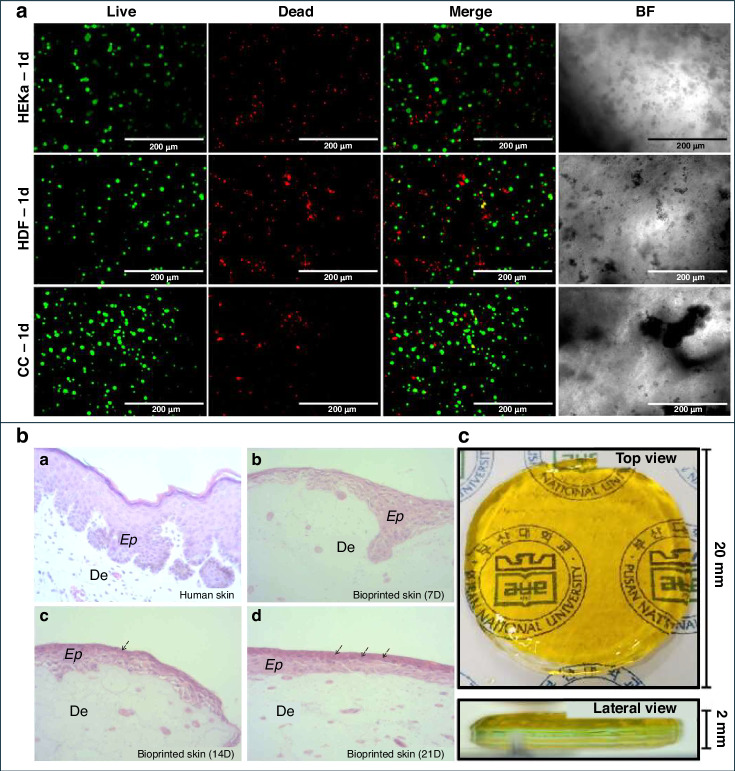
3D Bioprinted HSEs and key advantages. **a** Representative constructs containing only human epidermal keratinocytes (HEKa), only human dermal fibroblasts (HDF), or a co-culture (CC) of both cell types, demonstrating the adaptability of the printing approach for different cellular compositions. **b** Hematoxylin and eosin (H&E) staining showing stratified epidermal layers and organized dermal compartments. **c** Schematic of the construct design illustrating sequential deposition of dermal and epidermal layers. They enable reproducible fabrication of full-thickness skin with physiological architecture, supports high cell viability and balanced proliferation in co-culture, and facilitates applications in drug efficacy/safety testing, cosmetic evaluation, and replacement of animal models through simplified and cost-effective production

Advanced 3D bioprinting techniques are defined as methods that allow high-resolution, multi-material printing with precise spatial control to reproduce layered skin structures while also enabling the addition of complex features such as vascular channels^71^, neural elements, and sebaceous appendages^72^. These innovations enhance the physiological relevance and translational potential of bioprinted HSEs for applications in wound healing^73^, grafting^63^, cosmetic testing^74^, aging^75^, and preclinical drug evaluation^76–78^. Recent studies have further integrated patient-derived cells, enabling personalized skin models for studying genetic disorders or individualized therapeutic responses. Complementary imaging and characterization tools, including multiphoton microscopy and Raman spectroscopy, validate the morphological and biochemical fidelity of these constructs, ensuring that bioprinted HSEs meet the functional benchmarks required for advanced in vitro skin models^67,79–82^ (Fig. 4).

#### Skin organoids

Skin organoids represent a significant advance in in vitro skin research, offering 3D, multicellular systems that closely mimic the structure and function of human skin. Often derived from stem cells or reaggregated primary cells, these organoids are cultivated through precise differentiation protocols that simulate embryonic development^83^, using specific growth factors and tailored culture conditions. This approach enables the formation of stratified squamous epithelium, marking a pivotal advance in skin biology and facilitating the development of sophisticated 3D skin models^84^. Today, skin organoids serve as invaluable tools for disease modeling, compound screening, and regenerative medicine.

### Potential applications of skin organoids in personalized modeling

Skin organoids enable the development of personalized models by leveraging patient-derived or genetically modified cells, offering genetically relevant platforms for studying skin biology and therapeutic responses. These systems provide enhanced fidelity in disease modeling through three core features: (i) the ability to recapitulate the 3D architecture of skin, including appendages such as hair follicles and sebaceous glands; (ii) the use of patient-derived or genetically modified cells for personalized and genetically relevant disease modeling; and (iii) the capacity for long-term culture, supporting studies on chronic disease progression, regenerative processes, and sustained drug responses^85^. These models capture individual-specific genetic backgrounds, making them powerful tools for investigating inherited disorders, patient-specific immunopathology, and tailored treatment strategies^86,87^. Recent advances have further improved their biological relevance by incorporating immune cells such as Langerhans cells and T-cells^88^, enabling the modeling of complex inflammatory skin diseases, including psoriasis, eczema, and allergic contact dermatitis. Notably, human induced pluripotent stem cells (iPSC)-derived organoids have demonstrated the formation of a functional DEJ, where basal keratinocytes adhere to a laminin-332 and collagen IV-rich basement membrane via type I hemidesmosomes and integrin β1-based adhesion complexes, closely mimicking native skin histology.

The inclusion of skin appendages allows organoids to model previously inaccessible conditions, such as alopecia^89^ and acne, which depend on follicular and sebaceous gland biology^90,91^. Furthermore, using patient-derived cells introduces a layer of personalization, making organoids powerful tools for studying inherited skin disorders and personalized therapeutic responses^92^. This is especially relevant when immune components are included, enabling the investigation of patient-specific immunopathology and responses to immunomodulatory treatments^93^. Additionally, the extended culture longevity of skin organoids facilitates long-term studies, making them valuable for modeling chronic skin disease, wound healing, and regeneration. Their complex 3D structure supports drug penetration and metabolism studies across multiple layers, improving the translational relevance of preclinical testing.

### Technological innovations and methodological enhancements

Recent advances in skin organoid research have given rise to three distinct categories of organoids: (i) basic skin layer organoids, (ii) appendage-enhanced organoids, and (iii) disease-specific organoids. Each type contributes uniquely to skin biology, regenerative medicine, and practical applications such as safety assessment.

**Fig. 5:**
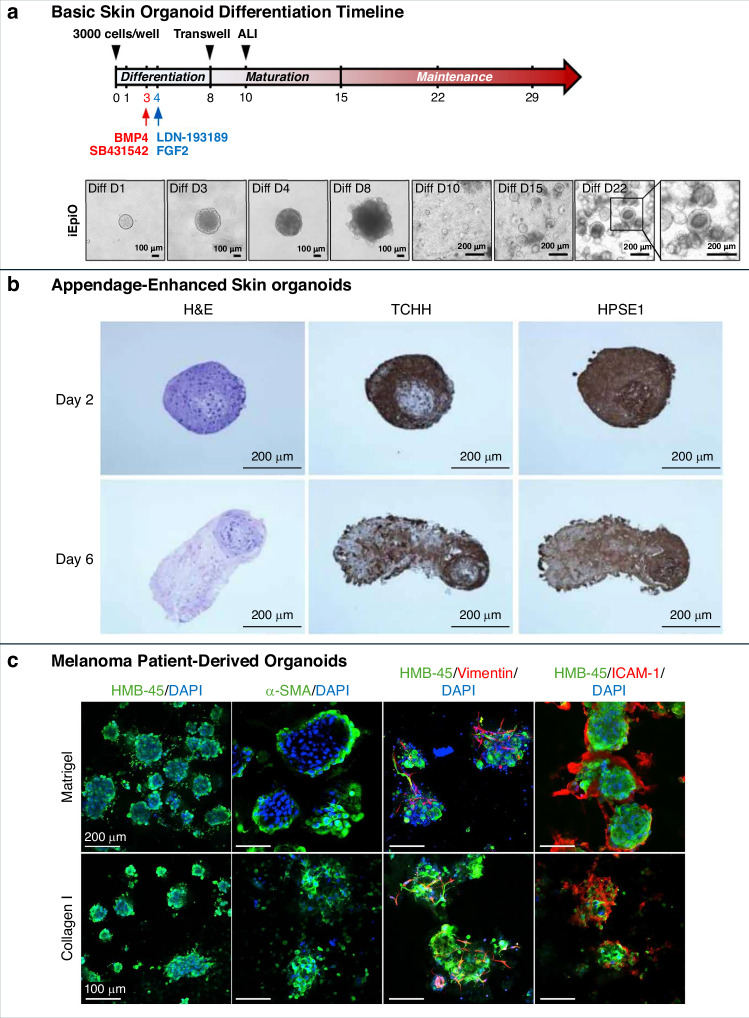
Advances in skin organoid models: from basic epidermal layers to appendage formation and disease-specific applications. **a** Basic Skin Layer Organoids: Timeline and process of epidermal organoid differentiation from induced pluripotent stem cells (iPSCs). **b** Appendage-Enhanced Organoids: Histological and immunohistochemical analyses of skin organoids at Day 2 and Day 6, showing the expression of markers such as H&E, TCHH, and HPSE1. **c** Disease-Specific Organoids: Confocal microscopy images of melanoma patient-derived organoids cultured in Matrigel and Collagen I, displaying marker expression, including HMB-45, α-SMA, Vimentin, and ICAM-1. Images reprinted from references^96,108,179^ with permission

Basic skin layer organoids, typically derived from iPSCs, replicate essential skin compartments, including stratified epidermal and dermal structures. These organoids allow researchers to study key biological processes such as epidermal differentiation, barrier formation, and wound healing in a physiologically relevant 3D setting^94–96^ (Fig. 5a). Long-term culture systems support adult epidermal stem cell maintenance and basal-apical polarity, making these organoids particularly useful for studying tissue regeneration and homeostasis^97^.

Appendage-enhanced organoids represent a major step forward in physiological relevance. They contain structures such as hair follicles^98–101^ and sebaceous glands^90,102^, which are formed through self-organizing epithelial–mesenchymal interactions that recapitulate embryonic skin development. In iPSC–derived models, signaling pathways including Wnt, BMP, and FGF are modulated to guide follicle development. Dermal condensate formation supports follicle-like structures, while sebaceous glands arise through regionally guided differentiation^102^. The maturation of these appendages typically requires prolonged culture and staged media formulations, with early induction phase followed by maintenance in calcium- and lipid-rich environments^98^. These conditions promote the development of epidermal barrier functions and appendage morphogenesis. Although the reproducibility and full maturation of these structures remain challenges, the presence of features such as DEJ, neuronal innervation and hair follicle cycling enhances their potential for practical testing and mechanistic studies^89^. This category of organoids has facilitated research into conditions such as alopecia and acne and offers utility for assessing the effects of cosmetic and dermatological products^90,99,100^.

Emerging protocols for generating iPSC-derived keratinocytes, fibroblasts, and endothelial cells are playing an increasingly important role in improving the reproducibility and personalization of skin organoid platforms. Directed differentiation methods now allow for the production of lineage-specific cell types with defined phenotypic markers and functional characteristics. These iPSC-derived cells offer several advantages over primary or immortalized lines, including reduced donor-to-donor variability, the ability to derive matched cell types from a single genetic background, and scalability for high-throughput applications^103,104^. iPSC-based approaches also enable patient-specific modeling for rare skin disorders and allow for the study of genetic risk factors, sex differences, and ethnic variability in skin biology^105–107^. As protocols for differentiation and maturation continue to improve, iPSC-derived components are expected to become foundational for creating more standardized, regulatory-compatible skin models across both organoid and MPS platforms.

Disease-specific skin organoids enable targeted modeling of skin pathologies using patient-derived or genetically modified cells, allowing researchers to reproduce disease-specific phenotypes and cellular responses. These models typically consist of multiple cell types relevant to human skin, including keratinocytes, fibroblasts, melanocytes, mesenchymal cells, and, in some cases, immune components such as Langerhans cells or T cells. For instance, melanoma patient-derived organoids (MPDOs) maintain intratumoral heterogeneity and immune responsiveness, making them suitable for evaluating immunotherapies and drug resistance^108,109^ (Fig. 5b). Additional models have been developed for conditions such as atopic dermatitis, SARS-CoV-2 and mpox viral infections, and bacterial colonization by Staphylococcus aureus^91,110,111^. These disease models are validated by the expression of lineage-specific markers and immune indicators, including HMB-45 for melanocytes, α-smooth muscle actin (α-SMA) for myofibroblasts, vimentin for mesenchymal cells, and ICAM-1 for inflammatory activity (Fig. 5c). The cellular diversity and structural fidelity of these organoids make them highly suitable for studying disease mechanisms, screening compounds, and evaluating therapeutic responses in a human-relevant context.

### Implications for regulatory science and cosmetic industry

As skin organoid technology advances, its integration into regulatory frameworks, such as those of the OECD and the FDA, has become a critical consideration^112^. Organoid-based assays hold great promise for safety evaluation and skin sensitization testing, providing ethically responsible alternatives to animal models and accelerating product development in the cosmetic industry^113^. For instance, patient-derived organoids can be used to screen compounds for allergenic or irritant potential, offering higher translational fidelity than conventional 2D cultures.

However, significant challenges remain before organoids can serve as diagnostic standards or gain widespread regulatory acceptance. Skin organoids often exhibit structural heterogeneity, batch-to-batch variability, and incomplete recapitulation of in vivo physiology, particularly in vascularization, immune cell distribution, and appendage maturation. These limitations hinder reproducibility and complicate the interpretation of organoid-based assays for clinical or regulatory decision-making. Moreover, their microenvironment and mechanical properties may differ from those of native skin, affecting barrier function and long-term stability. To move toward FDA-approved applications, standardization and quality control are essential. Strategies include implementing robust biomanufacturing pipelines, defining acceptance criteria for organoid size, morphology, and functional markers, and integrating high-content imaging and molecular profiling for batch validation. Hybrid approaches that combine organoids with MPS (e.g., perfused organoid-on-chip platforms) can improve vascularization, nutrient delivery, and functional reproducibility, increasing their suitability for preclinical and regulatory use^114^. Additionally, incorporating patient-derived cells with defined genetic and immunological profiles may enhance their predictive power for personalized safety assessments.

Looking forward, the cosmetic and pharmaceutical industries could leverage validated organoid platforms for skin irritation, sensitization, and permeability testing under OECD and FDA guidelines, ultimately reducing reliance on animal testing. By addressing heterogeneity, improving physiological fidelity, and adopting standardized manufacturing practices, skin organoids can progress from research tools to reliable platforms for regulatory toxicology and translational applications.

### Skin-on-a-chip (SoC)

OoC technology represents a major advancement in tissue modeling by enabling the recreation of organ-level functions within microfluidic platforms^115^. These systems use microscale channels and chambers to culture human cells under dynamic conditions that closely mimic the in vivo environment^116–120^. By incorporating mechanical stimulation, fluid flow, and spatial organization, OoC devices offer more physiologically relevant models than traditional static cultures^121,122^. Over the past decade, OoC platforms have demonstrated their scientific value across a wide range of research fields, including disease modeling, pharmacokinetics, and toxicity testing. Their ability to recapitulate human physiology in vitro has been extensively validated in academic studies. Now, the field is undergoing a clear transition from research innovation to industrial application. This shift reflects the growing technical maturity of the platform, along with increased demand for standardized, robust, and reproducible systems that are compatible with regulatory and commercial workflows.

Recent developments have focused on off-the-shelf availability, modularity, and integration with real-time analytical tools. Many platforms now support continuous monitoring of tissue responses, are compatible with automated liquid handling, and offer scalable formats suitable for high-throughput applications. These advances are driving the industrialization of OoC, positioning it as a practical and reliable solution for pharmaceutical development, cosmetic safety evaluation, and next-generation toxicology. As a result, OoC is no longer viewed solely as a research tool but as a commercially viable and regulatory-relevant technology platform.

### Dynamic SoC platforms

Building on the advantages of OoC technology, the SoC model has emerged as an innovative and highly efficient platform for skin research. SoC model emulates the physiological environment of human skin by integrating microfluidic flow, mechanical cues, and compartmentalized tissue organization. Unlike static Transwell cultures, SoC platforms support dynamic fluid flow, which facilitates continuous nutrient delivery and waste clearance, mimicking capillary exchange in vivo. Typically, SoC systems operate at flow rates ranging from 1 to 100 μL/min, depending on channel geometry and desired shear stress levels. For example, flow rates of 5–10 μL/min in microchannels (width: ~500 μm; height: ~200 μm) can generate shear stresses in the range of 0.01–0.1 dyne/cm², which are comparable to those experienced by dermal microvascular networks. These dynamic conditions help preserve cell viability, promote epidermal-dermal differentiation, and enhance barrier integrity. Peristaltic pumps (e.g., Ismatec REGLO) or gravity-based tilting platforms (e.g., CellASIC ONIX or manually adjustable rocker systems) are commonly used to generate pulsatile or unidirectional flow, adding physiological relevance through cyclic mechanical loading^123^. In addition, dynamic stimulation enhances the functional readout of SoC systems. For instance, under perfusion culture, transepithelial electrical resistance (TEER) values of over 1000 Ω cm^2^ have been achieved, indicating strong barrier formation and tight junction maturation. Some platforms also enable cyclic strain or interstitial flow across multilayer constructs, mimicking mechanical stress associated with wound healing or topical application of cosmetics.

These dynamic features not only improve biological fidelity but also allow real-time monitoring through integrated electrodes, optical windows, and live-cell imaging modules. This functional versatility has positioned SoC models as a practical and scalable alternative to more complex 3D bioprinting and organoid systems. SoC platforms are now routinely used to evaluate chemical irritants, skin sensitizers, or therapeutic candidates in dermatology and cosmetics, offering reliable human-relevant readouts under controlled, reproducible conditions^124–128^.

### Limitations of traditional models and the advantages of SoC

Traditional models for evaluating skin and ocular irritation, such as animal testing and the Hen’s Egg Test on the Chorioallantoic Membrane (HET-CAM) assay, face major limitations in physiological relevance, mechanistic insight, and standardization. The HET-CAM assay involves applying test substances to the vascular membrane of a fertilized chicken egg and scoring irritation based on visible effects such as hemorrhage, coagulation, or lysis. While it avoids the use of live animals and is accepted for screening both dermal and ocular irritants, it lacks key human-specific features such as stratified epithelium, immune function, and a defined barrier structure. Moreover, its qualitative scoring method introduces subjectivity and reduces inter-laboratory reproducibility^129,130^.

SoC platforms present a promising alternative by incorporating human-derived cells into perfused microenvironments that better replicate in vivo skin and ocular physiology. For skin applications, SoC systems allow dynamic nutrient delivery, waste removal, and integration of immune or stromal components. These features improve tissue viability, enable long-term culture, and support real-time monitoring of key functional endpoints such as barrier integrity, cytokine secretion, and cell viability. Similarly, SoC-based ocular models are beginning to emerge as replacements for static systems like RhCE and functional assays like HET-CAM. By mimicking corneal tissue structure within a controlled, fluidic environment, ocular-on-a-chip platforms can offer more consistent, quantitative, and clinically relevant outputs for eye irritation assessment.

In contrast to conventional assays that rely on visual scoring or single-timepoint measurements, SoC platforms support continuous, quantitative analysis with greater reproducibility and reduced operator bias. They also eliminate the need for external transfers during barrier or permeability assays, streamlining workflows and reducing error. These advantages make SoC systems highly attractive for replacing legacy tests like HET-CAM in both skin and ocular toxicity evaluation. Despite significant progress in SoC, ocular MPS remains at an earlier stage of development. While the RhCE model is commonly used, it lacks perfusion and immune components and offers a limited dynamic response. The field still faces challenges in achieving standardized, high-throughput ocular MPS platforms suitable for widespread regulatory adoption. Nonetheless, emerging efforts in this area signal an important step toward replacing traditional ocular assays with more physiologically relevant and human-centered technologies.

### Development of scalable SoC models

Tissue engineering has advanced significantly in recent years, enabling the development of SoC platforms that overcome many limitations of traditional static or manually fabricated models. Earlier methods, such as soft lithography or polydimethylsiloxane (PDMS)-based prototyping, provided design flexibility but often resulted in device-to-device variation and limited reproducibility^131^. These challenges have been particularly evident in inter-laboratory studies, where variability in fabrication and assay execution can result in over 25–30% coefficient of variation (CV) in barrier assays or permeability tests^132^.

To address these limitations, more recent SoC systems have adopted advanced fabrication techniques that enhance reproducibility, throughput, and device standardization. As detailed in Table 2, the field has undergone a clear transition across four major fabrication modes:

**Table 2:** Evolution of microfluidic fabrication technologies

Fabrication methods	Chip materials	Cell culture and ECM composition	Key applications	Innovative features	Refs.
Soft lithography and replica molding	PDMS	HMVEC-ad, EGM-2MV, type 1 rat tail collagen	- Exploration of capillary morphogenesis and endothelial cell behavior - Creation of gradients, surface shear, interstitial flow, and enabling real-time imaging - Applications in angiogenesis, tumor metastasis, and immune response modulation	- Integration of 3D scaffolds and microfluidic networks within novel microfluidic devices - Enhanced precision in controlling the fluidic microenvironment - Advanced capabilities for real-time cellular dynamics monitoring - High-resolution imaging for detailed study of capillary morphogenesis and cellular processes	^175^
SLP 3D printing	PDMS	HUVECs, NHLFs, HEKn, cells	- Complex 2D and 3D structural fabrication in PDMS - Development of microfluidic devices with integrated sensors - Customizable organ-on-a-chip devices for diverse technological applications	- Enhanced digital patterning of PDMS for superior surface quality - Facilitates rapid prototyping with significant reductions in time and complexity - Automated, monolithic fabrication of specialized biochips like vasculature-on-a-chip and skin-on-a-chip	^176^
DLP 3D printing	Various resins (flexible, rigid, water-soluble, fluorescent, phosphorescent, conductive with PEDOT or copper nanoparticles), PDMS		- Development of microfluidic devices featuring integrated sensors - Production of complex multi-material objects for sensor applications - Integration of electrodes in microchip electrophoresis and conductive tracks in microfluidic devices for electrochemical detection	- State-of-the-art multi-material 3D printing for intricate microfluidic device fabrication - Real-time monitoring of resin exchange to prevent cross-contamination - Capability to fabricate microfluidic channels with minimal dimensions of 43 μm - Integration of conductive tracks for enhanced sensor functionality	^177^
Injection-molded plastic array 3D culture (IMPACT) platform	Polystyrene (PS), Polycarbonate (PC) films coated with pressure-sensitive adhesive (PSA)	HUVECs, LFs, Fibrinogen solution mixed with thrombin for 3D fibrin gels	- High-throughput 3D co-culture methodologies - Investigation of angiogenesis and vasculogenesis - Development of vascularized MPS	- Capillary-guided flow enabling spontaneous patterning of cell-containing gels - Rapid gel patterning achieved within seconds - Scalable design optimal for high-content screening and commercial deployment - Seamless integration within standard 96-well plate formats	^178^

**Fig. 6:**
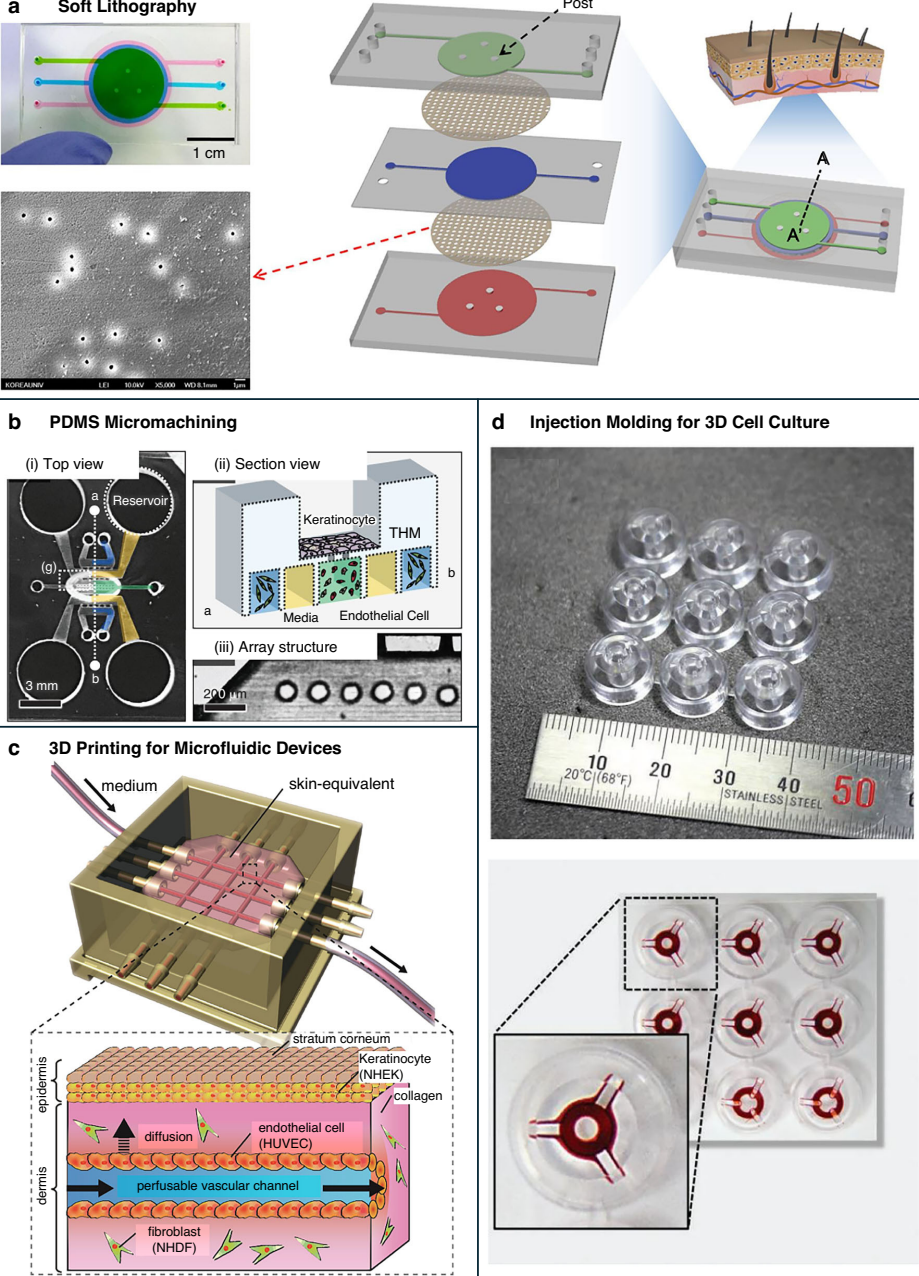
Fabrication technologies for SoC systems. **a** Soft lithography technique for creating microchannels in PDMS layers to simulate the layered structure of skin, allowing for the integration of cellular components and drug testing applications (adapted from ref. ^180^). **b** PDMS micromachining method: (i) Top view of device containing microfluidic reservoirs for SoC models. (adapted from ref. ^176^). (ii) Section 467 view—Schematic of keratinocyte culture showing co-culture with endothelial cells to recreate the skin barrier. 468 (iii) Array structure—SEM image demonstrating the precise microchannel structures fabricated in PDMS (**c**) 469 3D printing of skin models to produce complex skin structures incorporating perfusable vascular channels for 470 improved nutrient transport and drug delivery (adapted from ref. ^71^). **d** Injection molding for microfluidic 471 devices used in the production of highly scalable and reproducible systems with precise control over 472 vascularization and cellular environment (adapted from ref. ^181^)

(j) *Soft lithography*: Soft lithography remains a cornerstone in SoC device fabrication, enabling the production of intricate and high-resolution microstructures. Photolithography is employed to create a master mold, which is subsequently used to cast polydimethylsiloxane (PDMS) and form microfluidic channels^115^. While this method offers sophisticated design capabilities and a biocompatible environment, recent advances in mold design and fabrication processes have significantly improved reproducibility and scalability across different batches.^116–119^ (Fig. 6a).

(ii) *PDMS micromachining:* PDMS micromachining, such as successive laser pyrolysis (SLP), has been developed to address certain limitations of soft lithography^112^. SLP utilizes a continuous-wave laser to induce photothermal pyrolysis of PDMS, creating silicon carbide (SiC) patterns with high precision. This technique provides fine control over the depth and aspect ratio of microstructures, which is critical for accurately simulating the microenvironment of human skin (Fig. 6b).

(iii) *3D Printing for microfluidic designs:* 3D printing has emerged as a transformative technology for the fabrication of microfluidic devices, offering unparalleled design flexibility^120,121^. Techniques such as stereolithography (SLA), fused deposition modeling (FDM), and digital light processing (DLP) enable precise control over material deposition. This precision facilitates the creation of sophisticated SoC models that can serve as both casting molds and direct platforms for cell culture^122^ (Fig. 6c).

(iv) *Injection molding for 3D cell culture*: Injection molding represents the most scalable approach, capable of producing thousands of thermoplastic chips per day with dimensional tolerances below 5%^133,134^. The IMPACT platform exemplifies this trend, combining capillary-guided flow and cell-patterning strategies within standard 96-well plates, optimizing both scalability and physiological relevance^123–125^ (Fig. 6b).

This progression, as summarized in Table 2, reflects the field’s growing emphasis on industrial scalability, precision engineering, and application-specific innovation. As a result, SoC technology is advancing from lab-scale prototypes toward regulatory adoption and commercial deployment. These advances enhance model fidelity, throughput, and reproducibility, establishing SoC platforms as practical tools.

### Epidermis and dermis in SoC models

SoC platforms offer an advanced representation of skin layer composition, by integrating innovative fabrication strategies, diverse biomaterials, dynamic perfusion, and multicellular co-culture. These systems range from simplified epidermal constructs to full-thickness designs that incorporate dermal fibroblast, and vascular components, and support for air-liquid interface (ALI) conditions. A notable advancement is the use of fibrin-based dermal matrices within microfluidic chips, which—when combined with perfusion and controlled microenvironmental cues—can support improved epidermal development, stratification, and barrier function. Under ALI conditions, these systems have shown sustained tissue viability and morphological stability over extended culture periods. To support complex co-cultures involving keratinocytes, fibroblasts, endothelial cells, and pericytes, dual-compartment media strategies are often employed. These designs use low-calcium, serum-free media apically to promote keratinocyte differentiation under ALI, while basal channels supply endothelial-specific growth factor to maintain vascular networks. Compartmentalized flow systems approach allows for tailored support of each tissue layer, while flow regulation ensures adequate nutrient exchange and structural maintenance over prolonged culture periods^135^. The SoC models are composed of multilayered structures, including permeable membrane that allow active interactions between culture medium and cells. Dynamic perfusion ensures a continuous nutrient supply and waste removal, essential for developing and maintaining a functional skin barrier. This design supports high-throughput, automated testing, providing a cost-effective alternative to animal studies for drug screening and toxicological assessments. Collagen contraction rates, scaffold thickness, and cell density are optimized in these models to simulate structural integrity and functionality, with assessment methods like TEER and immunohistochemistry used to evaluate cellular barrier function^136^.

However, despite these advantages, it is important to recognized that replicating fully stratified, mature epidermal layers in SoC models remains technically challenging. While ALI culture within SoC platforms enhances barrier formation, hydrogel-based dermal matrices and perfused microenvironments may not inherently support the clear stratification typically achieved in static Transwell-based HSEs. HSEs, which have long used ALI culture on membrane inserts, often demonstrate well-organized basal, spinous, granular, and cornified layers. In contrast, SoC systems require careful optimization of flow rate, ECM composition, and keratinocyte density to approach similar levels of tissue stratification and differentiation. Moreover, current SoC platforms often lack standardized protocols for these parameters, and variability in commercial keratinocyte lines (e.g., NHEKs, HaCaTs) further complicates reproducibility.

**Fig. 7:**
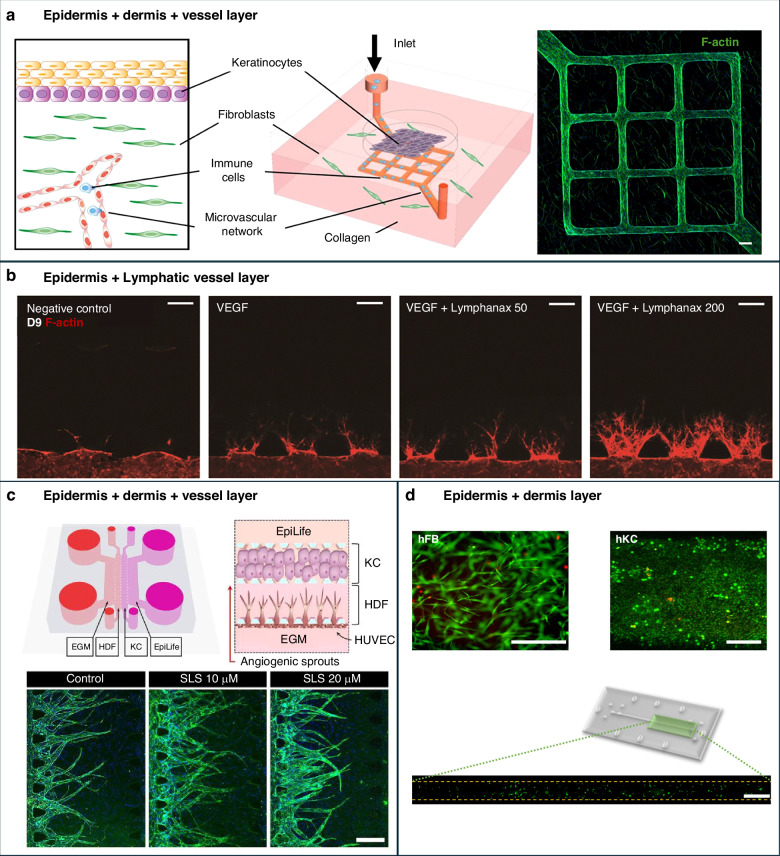
Innovations in SoC systems. **a** Schematic representation of an SoC platform designed to model HSV infection, incorporating keratinocytes, fibroblasts, immune cells, and a microvascular network within a collagen matrix. This system replicates the native skin environment and facilitates the evaluation of antiviral drug efficacy. **b** Microfluidic device for studying lymphangiogenesis in a 3D co-culture system of skin and lymphatic tissues, enabling the analysis of lymphatic vessel formation under various experimental conditions. **c** Skin irritation testing platform utilizing a microfluidic system that co-cultures keratinocytes, fibroblasts, and HUVEC. This system simulates the layered structure of human skin and provides insights into skin irritation responses. **d** Microfluidic system employing Consecutive Layers Stacking (CLS), which enhances structural integrity and functionality by incorporating materials such as polymethyl methacrylate(PMMA) and PC membranes. This design addresses limitations of conventional PDMS-based systems for advanced skin modeling applications. Images reprinted from refs. ^119,120,137,182^ with permission

To address challenges in delivering physiologically relevant flow dynamics to both the epidermal and dermal compartments, a novel microfluidic platform has been introduced that features bilayered skin tissue and programmable flow control via syringe pumps^137^ (Fig. 7). This system establishes distinct apical and basal compartments, allowing directional nutrient delivery and more accurate replication of physiological conditions. In addition to syringe pump-based designs, other dynamic flow strategies have also been integrated into SoC models to replicate interstitial perfusion and regulate shear stress. For instance, peristaltic pumps can generate pulsatile or continuous flow at rates around 2–3 mL/h, supporting vascular perfusion and promoting epidermal barrier development^71^. Hydrostatic pressure-driven platforms, which rely on gravitational height differences between reservoirs, offer a pump-free and scalable alternative for achieving continuous perfusion, particularly in parallelized or low-resource systems^138^. Each approach offers specific advantages and limitations in terms of mechanical stress, control precision, and system complexity, and should be selected based on the intended application and desired degree of physiological mimicry. Despite ongoing advances, most SoC platforms still lack a fully mature DEJ with complete anchoring fibrils, and their layering outcomes remain inconsistent across systems. Although fibrin-based dermal matrices with dynamic perfusion can enhance epidermal morphogenesis and barrier function, these effects are model-specific and not yet standardized across laboratories. Continued comparative evaluation against established HSEs is essential to benchmark epidermal fidelity.

### Advances in vascularized and hybrid SoC models

One advantage SoC has over traditional evaluation platforms is its ability to develop biomimetic skin tissues that incorporate vascular structures. This vascularization is crucial for creating skin models that closely mimic human physiology, making them highly suitable for disease modeling and drug evaluation. By integrating endothelial cells to form vascular networks, these models enable studies on angiogenesis, a fundamental process in wound healing and various skin diseases^118^.

The development of vascularized skin models is particularly valuable for studying skin diseases that involve blood vessel dysfunction or abnormal growth. For example, vascularized SoC models offer insights into psoriasis, where increased blood flow and vessel formation contribute to the inflammation and thickening of skin layers. They also provide a powerful tool for researching rosacea, a condition linked to abnormal vascular responses that result in persistent redness and visible blood vessels. Moreover, the vascular networks in SoC models make them ideal for studying cutaneous lupus erythematosus, where the immune system attacks on blood vessels lead to skin damage, and hemangiomas, benign tumors formed by abnormal blood vessel growth, allowing researchers to observe disease progression and test therapies in a controlled setting.

Hybrid SoC models, which combine skin with additional cell types such as nerve or liver cells, extend the platform’s versatility for comprehensive toxicological assessments, including sensory irritation and hepatotoxicity. For instance, co-culturing human neural stem cells (hNSCs) and iPSC-derived hepatocyte-like cells (hiPSC-HEPs) within a single microfluidic platform allows for detailed simulations of cellular interactions, providing insights into systemic effects on skin.

### Incorporating immune and vascular and nerve components

Integrating immune and vascular components into SoC models marks a critical advance in dermatological research, especially for understanding complex immune-mediated skin conditions. This is particularly relevant for studying skin sensitization, which involves an overactive immune response to allergens and can lead to conditions such as contact dermatitis. Traditional sensitization assays, often relying on animal testing, provide limited mechanistic insight and raise ethical concerns. Recently, OECD-approved methods such as the “regional lymph node test method using flow cytometry” developed by the Korea Food and Drug Safety Evaluation Institute (KFDA) have begun shifting the field toward more human-relevant, mechanistically informative assays^21,139,140^.

SoC models now incorporate immune cells like Langerhans cells, dendritic cells, and macrophages to better emulate immune responses observed in human skin. These immune components influence the behavior of surrounding skin cells through cytokine-mediated signaling. For instance, pro-inflammatory cytokines like IL-1, IL-6, and TNF-α modulate keratinocyte proliferation and differentiation, while also regulating fibroblast-mediated ECM remodeling through altered collagen synthesis^141^. These immune-epithelial interactions are modeled in SoC systems through co-culture strategies, where immune cells are embedded within microfluidic compartments alongside keratinocytes or fibroblasts, or seeded in adjacent chambers connected via perfusion channels to simulate paracrine signaling. Alternatively, immune cell-conditioned media collected from upstream immune-primed SoC units can be perfused through downstream skin constructs, allowing controlled exposure to cytokine-rich environments. These modeling strategies are particularly useful for investigating inflammatory skin disorders such a psoriasis, allergic dermatitis, and wound healing responses^142^. Importantly, the inclusion of immune cells enhances the functional integrity of the model. In co-culture systems using keratinocytes (e.g., HaCaT) and monocyte-derived cells (e.g, U937), SoC models demonstrate improved barrier properties, elevated expression of tight junction proteins such as ZO-1 and claudin-1, and sustained viability under ALI conditions. These features support more accurate modeling of in vivo skin physiology and response to topical exposures^143^. However, it is important to acknowledge that the integration of immune components remains technically variable and biologically incomplete across current SoC platforms. Reproducibility is constrained by batch-to-batch variation in immune cell sources and the absence of consensus activation protocols. These limitations mirror similar challenges found in HSEs, emphasizing that neither platform yet fully capture the dynamic immune complexity of native skin. To address these issues, future efforts may benefit from the use of iPSC-derived immune and skin cell populations, which offer greater standardization potential and patient-specific relevance. Additionally, the establishment of multi-site validated protocols for immune cell activation and co-culture could enhance reproducibility and accelerate adoption in regulatory contexts.

**Fig. 8:**
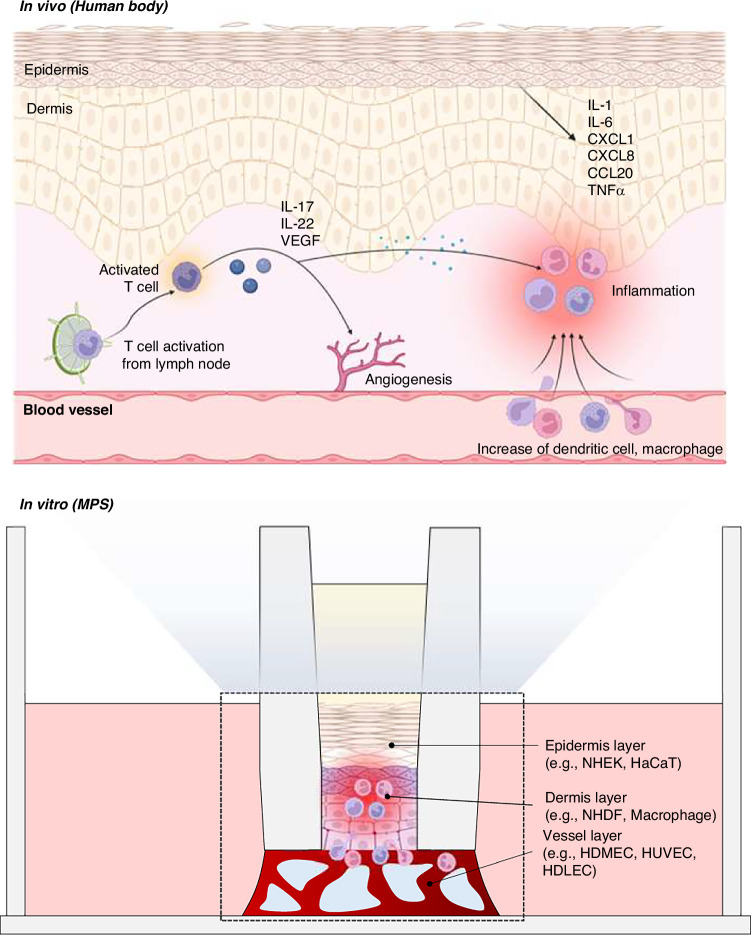
Schematic illustration of psoriatic immune responses and their modeling within skin MPS. In vivo, psoriatic inflammation is initiated by activated T cells that migrate from lymph nodes to the dermis and secrete cytokines such as IL-17, IL-22, and VEGF. These signals promote angiogenesis and stimulate keratinocytes to release additional pro-inflammatory mediators, including IL-1, IL-6, CXCL1, CXCL8, CCL20, and TNF-α. This cascade leads to immune cell recruitment, endothelial activation, and chronic inflammation. Skin MPS recapitulates these interactions using a stratified co-culture of keratinocytes (e.g., NHEK, HaCaT), fibroblasts (e.g., NHDF), endothelial cells (e.g., HDMEC, HUVEC, HDLEC), and immune cells (e.g., macrophages). This configuration enables the modeling of chronic immune–vascular crosstalk in inflammatory skin diseases such as psoriasis. Created with BioRender.com

Immune-vascular crosstalk in SoC platforms is enabled by perfuable vascular or lymphatic compartments that support immune cell trafficking and cytokine diffusion. Upon inflammatory stimulation, immune cells within these vascularized models initiate angiogenic signaling and endothelial activation, recapitulating key features of chronic skin diseases. For instance, in psoriatic SoC models, activated T cells drive angiogenesis and persistent inflammation via sustained cytokine secretion. Figure 8 summarizes this cascade, depicting T cell activation, dendritic cell recruitment, and cytokine-induced vascular remodeling^144^. Despite these promising advances, the degree of vascular fidelity in SoC platforms remains limited. Many models employ simplified or partially matured endothelial structures that do not fully mimic the hierarchical vascular organization or functional responsiveness observed in vivo. Moreover, the fluidic architecture of SoC systems, while enabling perfusion, can inadvertently oversimplify the dynamic gradients and mechanical cues crucial for immune-endothelial interactions. These oversimplifications may affect biological interpretation and regulatory extrapolation of toxicity or drug efficacy data derived from SoC model.

In addition to immune and vascular elements, the incorporation of nerve components is increasingly recognized as essential for mimicking the full physiological complexity of skin. Skin-nerve crosstalk plays a crucial role in sensing external stimuli and regulating immune responses^145,146^. Co-culture systems integrating sensory neurons and keratinocytes have demonstrated paracrine interactions that promote skin barrier integrity and induce expression of sensory receptors in the epidermis. These advanced models enable neurotoxicity testing and help investigate how chemicals or inflammatory states influence nerve function within the skin. However, most current systems are limited to exploratory proof-of-concept studies, and challenges remain in sourcing subtype-specific neurons, maintaining their long-term function, and integrating electrophysiological readouts into chip platforms.

While the incorporation of immune, vascular, and neural components has significantly advanced the field of skin MPS, current SoC platforms remain biologically immature and technically constrained. Many of the limitations noted in HSEs—such as incomplete layering, variable cell sourcing, and lack of dynamic multi-system interactions—persist in SoCs. These shared limitations underscore the importance of avoiding overstatement when positioning SoC as a superior alternative to existing models. Moving forward, the field would benefit from modular designs that allow standardized, interoperable integration of immune, vascular, and nerve systems, alongside validated protocols for cell preparation and readout calibration. Only through such advances can SoC platforms achieve the biological fidelity necessary for nonclinical regulatory use and personalized medicine applications.

### Advanced techniques for quantitative analysis of skin MPS

As skin MPS models grow in biological complexity with features like 3D co-culture, vascularization, and immune integration, advanced quantitative analysis tools have become essential for studying dynamic interactions in a standardized and scalable manner. While this section does not aim to provide a comprehensive list of all available tools, it presents selected representative techniques that are either widely used in related organoid or organ-on-a-chip systems or demonstrate strong applicability to skin MPS.

*AngioTool* is a widely used ImageJ plugin designed for analyzing vascular networks. It calculates parameters such as vascular area, vessel length, and branching index, which are critical for angiogenesis studies^147^. While AngioTool is user-friendly and reliable for small datasets, it requires manual adjustments that may introduce inconsistencies when analyzing larger datasets. *OrganoID* utilizes deep learning for high-throughput analysis by automating the identification, labeling, and tracking of organoids using convolutional neural networks (CNNs)^148^. It excels in handling large datasets and tracking dynamic changes. However, it remains dependent on traditional fluorescent staining methods, which can be time-consuming and costly. *NuSeT* is a tool specifically designed for efficient and precise vascular analysis. Leveraging advanced deep learning, it enables spatial analysis and real-time monitoring, making it ideal for studies focusing on angiogenesis and tissue regeneration^149^. NuSeT offers a comprehensive view of vascular networks, making it particularly beneficial for large-scale datasets. *Angio-Net* represents the latest innovation in quantitative analysis. By applying deep learning techniques, Angio-Net eliminates the need for traditional cellular staining^150^. Utilizing SegNet-based networks and conditional generative adversarial networks (cGANs), it transforms bright-field images into pseudo-fluorescent images. This capability allows for real-time, non-invasive studies of live cells, preserving cell viability and enabling researchers to observe dynamic processes in 3D co-cultures with high accuracy. *VONet* is a deep learning model developed for 3D reconstruction of organoid morphology. By employing a synthetic dataset of virtual organoids for training, VONet reconstructs complete 3D morphology from a limited number of focal plane images^151^. Unlike conventional methods that rely on extensive z-stacks, VONet offers rapid and accurate 3D modeling, even in deep focal regions where traditional imaging methods often lose detail. This innovation significantly enhances efficiency in high-content screening applications.

These tools demonstrate how recent advances in computer vision and machine learning can support the analysis of complex skin MPS. Although not all were originally developed for dermatological models, they offer adaptable capabilities that address current limitations in quantifying structural and functional aspect of skin MPS. In addition to vascular and morphological analysis, other quantitative techniques such as TEER measurements, automated immunostaining-based scoring, and image-based stratification analysis are increasingly used to evaluate barrier function and epidermal layer organization^135,152,153^. These metrics provide standardized, quantifiable endpoints that help overcome reproducibility and validation gaps in current MPS platforms.

### Skin MPS-based disease modeling

Skin MPS platforms have emerged as powerful tools for modeling complex dermatological diseases in a physiologically relevant and scalable manner. By incorporating patient-derived cells, vascular and immune components, and stratified architectures, these platforms enable accurate recapitulation of pathological features observed in vivo. Below, we highlight representative use cases across major disease areas.

#### Psoriasis

Psoriasis is characterized by keratinocytes hyperproliferation, immune infitration, and vascular remodeling. In vivo, psoriatic lesions show thickened epidermis with elongated rete ridges and tortuous blood vessels, driven by pro-inflammatory cytokines (e.g., TNF-α, IL-23, IL-17). Vascularized SoC models allow real-time monitoring of angiogenesis and immune-keratinocyte interactions under cytokine-rich conditions. Incorporating T cells or macrophages within these plarform has enabled the simulation of inflammatory cascades and tissue responses that mirror clinical psoriatic pathology^154,155^.

#### Atopic dermatitis (AD)

**Fig. 9:**
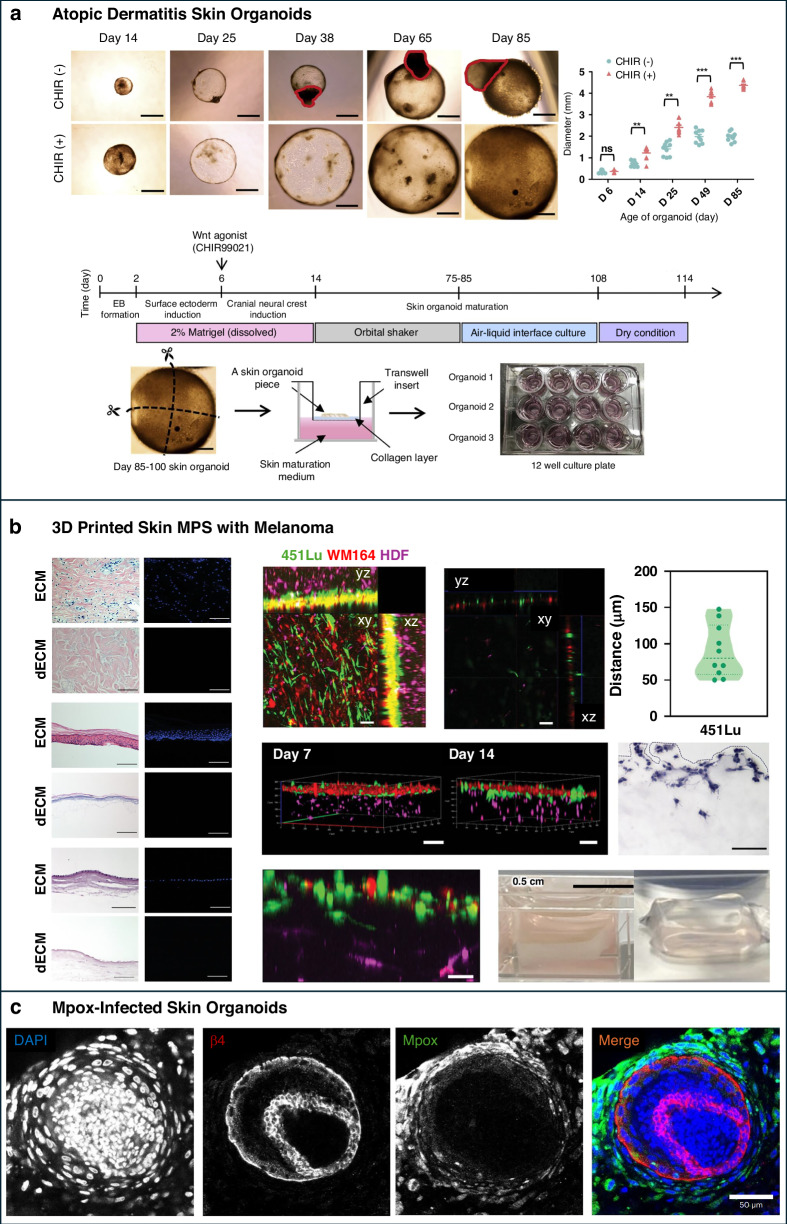
Advances in skin MPS-based disease modeling. **a** Timeline and culture protocol for generating skin organoids to model AD, showcasing key stages of development and maturation. **b** Confocal and histological analyses of melanoma cell invasion within a 3D-printed skin MPS, highlighting cellular interactions and invasion dynamics. **c** Human skin organoid model of mpox infection, demonstrating viral replication in keratinocytes, with integrin β4 and nuclear staining providing cellular context. Images reprinted from references^111,158,163^ with permission

Atopic dermatitis (AD) features a defective skin barrier, immune dysregulation, and microbial dysbiosis^156,157^. A notable model invovles ALI-cultured iPSC-derived skin organoids exposed to *Staphylococcus aureus*, a common skin pathogen in AD patients (Fig. 9a). These organoids exhibited reduced expression of filaggrin and loricrin, alongside elevated thymic stromal lymphopoietin and inflammatory cytokines, closely resembling lesional AD skin. Despite the absence of exogenous immune cells, epithelial-intrinsic responses captured halmark features of AD pathology, validating the use of this model for investigating host-microbe interactions and evaluating anti-inflammatory treatments^158^.

#### Melanoma

Melanoma progression is tightly linked to interactions between cancer cells, stromal cells, and the vascular niche^159^. SoC models integrating melanoma cells with dermal fibroblasts and endothelial cells enable dynamic obvservation of angiogenesis, metastasis, and drug response^160,161^. One study utilized a polyethylene terephthalate (PET) membrane-based layered chip to co-culture keratinocytes and melanoma cells for evaluating photodynamic therapy (PDT) (Fig. 9b)^162^. Upon activation with meso-tetraphenylporphyrin (TPP), PDT reduced melanoma viability, demonstrating the platform’s utility for therapeutic screening under controlled tumor microenvironment conditions^163^.

#### Monkeypox (Mpox)

Mpox presents with sequential skin lesion stages and is marked by viral replication within stratified skin layers, along with immune cell infiltration and vasculopathy^111^. An ALI-cultured iPSC-derived skin organoid model was infected with mpox virus and demonstrated expression of early and late viral genes (E3L, F13L), along with intracellular viral particle formation confirmed by TEM. The model faithfully reproduced viral lifecycle progrssion and epidermal disruption, and was used to evaluate tecovirimat, an antiviral agent that effectively reduced viral load. This example highlights the potential of MPS for studying emergin infectious skin diseases^164–167^.

Each of these disease-specific models successfully mimics in vivo phenotypes. These include inflammatory cytokine profiles, epithelial barrier loss, immune cell recruitment, angiogenic remodeling, and pathogen replication. Such features are reproduced through co-culture strategies, dynamic perfusion, and layered ALI systems. The use of patient-derived cells further enhances personalized modeling. As shown in Fig. 9, the integration of immune and vascular components enables physiologically relevant responses that are critical for dermatological research, drug screening, and therapeutic development.

### Conclusion and perspectives

The enactment of the FDA Modernization Act 2.0 and the Modernization of Cosmetics Regulation Act of 2022 (MoCRA) marks a pivotal shift in preclinical and cosmetic testing, encouraging the adoption of human-relevant and non-animal approaches. Within this regulatory evolution, skin MPSs have emerged as promising tools for predictive safety and efficacy assessment. These platforms provide physiologically relevant microenvironments that support mechanistic studies, long-term exposure evaluations, and patient-specific testing. As such, they align with the growing demand for alternatives to animal models and enable more translationally meaningful insights.

Despite these advances, current skin MPS remain biologically and technically incomplete in several aspects. The integration of vascular, immune, and neural components often lacks full functionality and coordination, limiting the systems’ ability to recapitulate in vivo skin dynamics. These biological gaps may constrain the interpretability of complex phenomena such as chronic inflammation, immune trafficking, or neurocutaneous interactions. In addition, the fluidic environments of SoC platforms can oversimplify native gradients and mechanical cues, which raises caution when extrapolating test results to human outcomes. Further limitations include incomplete vascular maturation, difficulty in achieving stable and physiologically active immune and neural integration, and restricted culture longevity for chronic toxicity or disease studies. The absence of standardized protocols, harmonized readouts, and validated quality metrics across laboratories continues to hinder reproducibility and broader regulatory acceptance.

To bridge these gaps, several actionable steps are needed: (i) establishing inter-laboratory validation protocols, (ii) developing reference materials and performance benchmarks, and (iii) revising OECD guidelines to incorporate organoid- and chip-based assays as standardized alternatives. Future research should prioritize enhancing long-term culture stability, integrating innervation to support neurocutaneous modeling, and expanding the use of iPSC-derived, patient-specific tissues to reflect inter-individual and sex-based biological variability. In particular, the development of iPSC-based isogenic skin MPS platforms offers a promising strategy for reducing donor-to-donor variability and improving experimental standardization. These models can provide genetically matched keratinocytes, fibroblasts, and endothelial cells that support reproducible co-culture, disease modeling, and patient-tailored therapeutic testing. Their scalability and consistency make them strong candidates for future regulatory frameworks.

Expanding the biological complexity of skin MPS must be pursued alongside scalability, automation compatibility, and cost-effectiveness. These priorities will accelerate the transition of MPS platforms from exploratory tools to validated, regulatory-grade testbeds. By combining technological innovation, regulatory alignment, and industrial collaboration—as exemplified by ongoing work with Kolmar Korea—skin MPSs are poised to become a cornerstone of next-generation preclinical evaluation. These platforms not only advance ethical and human-relevant testing but also hold transformative potential for precision medicine, cosmetic safety, and translational dermatology.

## References

[1] Zhu, Y. et al. Dynamic microphysiological system chip platform for high-throughput, customizable, and multi-dimensional drug screening. *Bioact. Mater.* **39**, 59–73 (2024). 38800720 10.1016/j.bioactmat.2024.05.019 PMC11127178

[2] Vuorenpää, H. et al. Building blocks of microphysiological system to model physiology and pathophysiology of human heart. *Front. Physiol.* **14**, 1213959 (2023). 37485060 10.3389/fphys.2023.1213959 PMC10358860

[3] Monteduro, A. G. et al. Organs-on-chips technologies – A guide from disease models to opportunities for drug development. *Biosens. Bioelectron.* **231**, 115271 (2023). 37060819 10.1016/j.bios.2023.115271

[4] Miller, C. P. et al. Therapeutic targeting of tumor spheroids in a 3D microphysiological renal cell carcinoma-on-a-chip system. *Neoplasia* **46**, 100948 (2023). 37944353 10.1016/j.neo.2023.100948 PMC10663960

[5] Mansouri, M., Lam, J. & Sung, K. E. Progress in developing microphysiological systems for biological product assessment. *Lab Chip* **24**, 1293–1306 (2024). 38230512 10.1039/d3lc00876b

[6] Ko, J. et al. Patient-derived microphysiological systems for precision medicine. Adv. *Healthc. Mater.* **13**, 2303161 (2024). 10.1002/adhm.202303161 PMC11469251 38010253

[7] McDermott, O. et al. The impact of Industry 4.0 on the medical device regulatory product life cycle compliance. *Sustainability* **14**, 14650 (2022).

[8] Ajalik, R. E. et al. Human organ-on-a-chip microphysiological systems to model musculoskeletal pathologies and accelerate therapeutic discovery. *Front. Bioeng. Biotechnol.* **10**, 846230 (2022). 35360391 10.3389/fbioe.2022.846230 PMC8964284

[9] Nam, U. et al. Microphysiological systems as organ-specific in vitro vascular models for disease modeling. *BioChip J*. 345–356 (2024).

[10] Morales, M. et al. Evaluation of fibrin-based dermal-epidermal organotypic cultures for in vitro skin corrosion and irritation testing of chemicals according to OECD TG 431 and 439. *Toxicol. Vitr.* **36**, 89–96 (2016). 10.1016/j.tiv.2016.07.010 27448499

[11] Dartt, D. A. & Willcox, M. D. P. Complexity of the tear film: importance in homeostasis and dysfunction during disease. *Exp. Eye Res.* **117**, 1–3 (2013). 24280033 10.1016/j.exer.2013.10.008 PMC4225770

[12] Schmook, F. P., Meingassner, J. G. & Billich, A. Comparison of human skin or epidermis models with human and animal skin in in-vitro percutaneous absorption. *Int. J. Pharm.* **215**, 51–56 (2001). 11250091 10.1016/s0378-5173(00)00665-7

[13] Verstraelen, S. et al. Improvement of the bovine corneal opacity and permeability (BCOP) assay as an in vitro alternative to the Draize rabbit eye irritation test. *Toxicol. Vitr* **27**, 1298–1311 (2013). 10.1016/j.tiv.2013.02.018 23501624

[14] OECD (2023), Test No. 498: In vitro Phototoxicity - Reconstructed Human Epidermis Phototoxicity test method, OECD Guidelines for the Testing of Chemicals, Section 4, OECD Publishing, Paris, 10.1787/7b2f9ea0-en.

[15] Duval, K. et al. Modeling physiological events in 2D vs. 3D cell culture. *Physiology* **32**, 266–277 (2017). 28615311 10.1152/physiol.00036.2016 PMC5545611

[16] Van Norman, G. A. Limitations of animal studies for predicting toxicity in clinical trials: is it time to rethink our current approach? *JACC Basic Transl. Sci* **4**, 845–854 (2019). 31998852 10.1016/j.jacbts.2019.10.008 PMC6978558

[17] Duarte, A. C. et al. Animal-derived products in science and current alternatives. *Biomater. Adv.* **151**, 213428 (2023). 37146527 10.1016/j.bioadv.2023.213428

[18] Arrowsmith, J. & Miller, P. Phase II and Phase III attrition rates 2011–2012. *Nat. Rev. Drug Discov.* **12**, 569–569 (2013). 23903212 10.1038/nrd4090

[19] Cook, D. et al. Lessons learned from the fate of AstraZeneca’s drug pipeline: a five-dimensional framework. *Nat. Rev. Drug Discov.* **13**, 419–431 (2014). 24833294 10.1038/nrd4309

[20] OECD (2010), Test No. 442A: Skin Sensitization: Local Lymph Node Assay: DA, OECD Guidelines for the Testing of Chemicals, Section 4, OECD Publishing, Paris, 10.1787/9789264090972-en.

[21] OECD (2010), Test No. 429: Skin Sensitisation: Local Lymph Node Assay, OECD Guidelines for the Testing of Chemicals, Section 4, OECD Publishing, Paris, 10.1787/9789264071100-en.

[22] Spielmann, H. et al. The ECVAM international validation study on in vitro tests for acute skin irritation: report on the validity of the EPISKIN and EpiDerm assays and on the skin integrity function test. *Altern. Lab. Anim.* **35**, 559–601 (2007). 18186667 10.1177/026119290703500614

[23] Alépée, N. et al. t4 Workshop report: State-of-the-art of 3D cultures (organs-on-a-chip) in safety testing and pathophysiology. *Altex* **31**, 441 (2014). 25027500 10.14573/altex1406111 PMC4783151

[24] Abaci, H. E. et al. Next generation human skin constructs as advanced tools for drug development. *Exp. Biol. Med.* **242**, 1657–1668 (2017). 10.1177/1535370217712690 PMC5786367 28592171

[25] Ataç, B. et al. Skin and hair on-a-chip: in vitro skin models versus ex vivo tissue maintenance with dynamic perfusion. *Lab Chip* **13**, 3555–3561 (2013). 23674126 10.1039/c3lc50227a

[26] Cubo, N. et al. 3D bioprinting of functional human skin: production and in vivo analysis. *Biofabrication* **9**, 015006 (2017). 10.1088/1758-5090/9/1/015006 27917823

[27] Zhang, X. & Zhang, Y. Tissue engineering applications of three-dimensional bioprinting. *Cell Biochem. Biophys.* **72**, 777–782 (2015). 25663505 10.1007/s12013-015-0531-x

[28] Zhang, S. et al. Convergence of 3D bioprinting and nanotechnology in tissue engineering scaffolds. *Biomimetics* **8**, 94 (2023). 36975324 10.3390/biomimetics8010094 PMC10046132

[29] Varaganti, P. & Seo, S. Recent advances in biomimetics for the development of bio-inspired prosthetic limbs. *Biomimetics* **9**, 273 (2024). 38786483 10.3390/biomimetics9050273 PMC11118077

[30] Ashammakhi, N. et al. Bioinks and bioprinting technologies to make heterogeneous and biomimetic tissue constructs. *Mater. Today Bio* **1**, 100008 (2019). 10.1016/j.mtbio.2019.100008 PMC7061634 32159140

[31] Chae, S., Ha, D.-H. & Lee, H. 3D Bioprinting strategy for engineering vascularized tissue models. *Int. J. Bioprinting* **9**, 748 (2023). 10.18063/ijb.748 PMC10370342 37502273

[32] Ma, Y. et al. Advancements of 3D bioprinting in regenerative medicine: exploring cell sources for organ fabrication. *Heliyon* **10**, e24593 (2024). 38318070 10.1016/j.heliyon.2024.e24593 PMC10838744

[33] Raees, S. et al. Classification, processing, and applications of bioink and 3D bioprinting: a detailed review. *Int. J. Biol. Macromol.* **232**, 123476 (2023). 36731696 10.1016/j.ijbiomac.2023.123476

[34] Wang, J., Cui, Z. & Maniruzzaman, M. Bioprinting: a focus on improving bioink printability and cell performance based on different process parameters. *Int. J. Pharm.* **640**, 123020 (2023). 37149110 10.1016/j.ijpharm.2023.123020

[35] Li, Z., Ruan, C. & Niu, X. Collagen-based bioinks for regenerative medicine: fabrication, application and prospective. *Med. Nov. Technol. Devices* **17**, 100211 (2023).

[36] Daikuara, L. Y. et al. 3D Bioprinting constructs to facilitate skin regeneration. *Adv. Funct. Mater.* **32**, 2105080 (2022).

[37] Freeman, F. E. & Kelly, D. J. Tuning alginate bioink stiffness and composition for controlled growth factor delivery and to spatially direct MSC fate within bioprinted tissues. *Sci. Rep.* **7**, 17042 (2017). 29213126 10.1038/s41598-017-17286-1 PMC5719090

[38] Karakaya, E. et al. Targeted printing of cells: evaluation of ADA-PEG bioinks for drop on demand approaches. *Gels* **8**, 206 (2022). 35448107 10.3390/gels8040206 PMC9032277

[39] Li, Z. et al. Tuning alginate-gelatin bioink properties by varying solvent and their impact on stem cell behavior. *Sci. Rep.* **8**, 8020 (2018). 29789674 10.1038/s41598-018-26407-3 PMC5964146

[40] Sharma, R. et al. 3D Bioprinting pluripotent stem cell derived neural tissues using a novel fibrin bioink containing drug releasing microspheres. *Front. Bioeng. Biotechnol.* **8**, 57 (2020). 32117936 10.3389/fbioe.2020.00057 PMC7026266

[41] Sekar, M. P. et al. Hyaluronic acid as bioink and hydrogel scaffolds for tissue engineering applications. *ACS Biomater. Sci. Eng.* **9**, 3134–3159 (2023). 37115515 10.1021/acsbiomaterials.3c00299

[42] Decante, G. et al. Engineering bioinks for 3D bioprinting. *Biofabrication* **13**, 032001 (2021). 10.1088/1758-5090/abec2c 33662949

[43] Pereira, R. F. et al. Bioprinting a multifunctional bioink to engineer clickable 3D cellular niches with tunable matrix microenvironmental cues. Adv. *Healthc. Mater.* **10**, 2001176 (2021). 10.1002/adhm.202001176 33135399

[44] Oliveira, H. et al. Extracellular matrix (ECM)-derived bioinks designed to foster vasculogenesis and neurite outgrowth: characterization and bioprinting. *Bioprinting* **22**, e00134 (2021).

[45] Gresham, R. C. H., Bahney, C. S. & Leach, J. K. Growth factor delivery using extracellular matrix-mimicking substrates for musculoskeletal tissue engineering and repair. *Bioact. Mater.* **6**, 1945–1956 (2021). 33426369 10.1016/j.bioactmat.2020.12.012 PMC7773685

[46] Muthukrishnan, L. Imminent antimicrobial bioink deploying cellulose, alginate, EPS and synthetic polymers for 3D bioprinting of tissue constructs. *Carbohydr. Polym.* **260**, 117774 (2021). 33712131 10.1016/j.carbpol.2021.117774

[47] Khoeini, R. et al. Natural and synthetic bioinks for 3D bioprinting. *Adv. NanoBiomed Res* **1**, 2000097 (2021).

[48] Soliman, B. G. et al. Stepwise control of crosslinking in a one-pot system for bioprinting of low-density bioinks. *Adv. Healthc. Mater.* **9**, 1901544 (2020). 10.1002/adhm.201901544 32323473

[49] GhavamiNejad, A. et al. Crosslinking strategies for 3D bioprinting of polymeric hydrogels. *Small* **16**, 2002931 (2020). 10.1002/smll.202002931 PMC7754762 32734720

[50] Choi, S., Ahn, H. & Kim, S.-H. Tyrosinase-mediated hydrogel crosslinking for tissue engineering. *J. Appl. Polym. Sci.* **139**, 51887 (2022).

[51] Naranjo-Alcazar, R. et al. Research progress in enzymatically cross-linked hydrogels as injectable systems for bioprinting and tissue engineering. *Gels* **9**, 230 (2023). 36975679 10.3390/gels9030230 PMC10048521

[52] Lee, J. et al. 3D Bioprinting using a new photo-crosslinking method for muscle tissue restoration. *npj Regen. Med.* **8**, 18 (2023). 37002225 10.1038/s41536-023-00292-5 PMC10066283

[53] Göckler, T. et al. Tuning superfast curing thiol-norbornene-functionalized gelatin hydrogels for 3D bioprinting. *Adv. Healthc. Mater* **10**, 2100206 (2021). 34145799 10.1002/adhm.202100206 PMC11481056

[54] Chen, L. et al. Touchable cell biophysics property recognition platforms enable multifunctional blood smart health care. *Microsyst. Nanoeng.* **7**, 103 (2021). 34963817 10.1038/s41378-021-00329-z PMC8651774

[55] Li, X. et al. Accurate modulation of photoprinting under stiffness imaging feedback for engineering ECMs with high-fidelity mechanical properties. *Microsyst. Nanoeng.* **8**, 60 (2022). 35669968 10.1038/s41378-022-00394-y PMC9163149

[56] Li, M. et al. 3D Bioprinting of heterogeneous tissue-engineered skin containing human dermal fibroblasts and keratinocytes. *Biomater. Sci.* **11**, 2461–2477 (2023). 36762551 10.1039/d2bm02092k

[57] Barros, N. R. et al. Biofabrication of endothelial cell, dermal fibroblast, and multilayered keratinocyte layers for skin tissue engineering. *Biofabrication* **13**, 035030 (2021). 10.1088/1758-5090/aba503 32650324

[58] Cao, H. et al. Current hydrogel advances in physicochemical and biological response-driven biomedical application diversity. *Signal Transduct. Target. Ther.* **6**, 426 (2021). 34916490 10.1038/s41392-021-00830-x PMC8674418

[59] Fang, W. et al. Hydrogels for 3D bioprinting in tissue engineering and regenerative medicine: Current progress and challenges. *Int. J. Bioprinting* **9**, 759 (2023). 10.18063/ijb.759 PMC10339415 37457925

[60] Teixeira, M. C. et al. 3D Bioprinting: an innovative technique for biofabrication applied to regenerative medicine and tissue engineering. in (eds Santana, M. H., Souto, E. B. & Shegokar, R.) Nanotechnology and Regenerative Medicine. Ch 9, 195–232 (Academic Press,2023).

[61] Jorgensen, A. M. et al. Multicellular bioprinted skin facilitates human-like skin architecture in vivo. *Sci. Transl. Med* **15**, eadf7547 (2023). 37792956 10.1126/scitranslmed.adf7547

[62] Olejnik, A. et al. 3D Bioprinting in skin related research: recent achievements and application perspectives. *ACS Synth. Biol.* **11**, 26–38 (2022). 34967598 10.1021/acssynbio.1c00547 PMC8787816

[63] Baltazar, T. et al. Three dimensional bioprinting of a vascularized and perfusable skin graft using human keratinocytes, fibroblasts, pericytes, and endothelial cells. *Tissue Eng. Part A* **26**, 227–238 (2020). 31672103 10.1089/ten.tea.2019.0201 PMC7476394

[64] Motter Catarino, C. et al. Incorporation of hair follicles in 3D bioprinted models of human skin. *Sci. Adv* **9**, eadg0297 (2023). 37831765 10.1126/sciadv.adg0297 PMC10575578

[65] Zhang, M. et al. Advances in 3D skin bioprinting for wound healing and disease modeling. *Regen. Biomater.* **10**, rabc105 (2022). 10.1093/rb/rbac105 PMC9845530 36683757

[66] Andrade, T. A. M. et al. 3D Bioprinting a novel skin co-culture model using human keratinocytes and fibroblasts. *J. Biomed. Mater. Res. Part A* **113**, e37831 (2025). 10.1002/jbm.a.37831 39487730

[67] Manita, P. G. et al. 3D Bioprinting of functional skin substitutes: from current achievements to future goals. *Pharmaceuticals* **14**, 362 (2021). 33919848 10.3390/ph14040362 PMC8070826

[68] Fernandes, S. et al. 3D Bioprinting: an enabling technology to understand melanoma. *Cancers* **14**, 3535 (2022). 35884596 10.3390/cancers14143535 PMC9318274

[69] Chai, R. J., Wong, W. L. & Beh, C. W. Developing a bioink for single-step deposition and maturation of human epidermis. Int. J. Bioprinting 9 (2023). 10.18063/ijb.738 PMC10261136 37323493

[70] Aleemardani, M. et al. The importance of mimicking dermal-epidermal junction for skin tissue engineering: a review. *Bioengineering* **8**, 148 (2021). 34821714 10.3390/bioengineering8110148 PMC8614934

[71] Mori, N., Morimoto, Y. & Takeuchi, S. Skin integrated with perfusable vascular channels on a chip. *Biomaterials* **116**, 48–56 (2017). 27914266 10.1016/j.biomaterials.2016.11.031

[72] Wang, Y. et al. Tailoring bioinks of extrusion-based bioprinting for cutaneous wound healing. *Bioact. Mater* **17**, 178–194 (2022). 35386443 10.1016/j.bioactmat.2022.01.024 PMC8965032

[73] Liu, Y. et al. 3D Bioprinting bioglass to construct vascularized full-thickness skin substitutes for wound healing. *Mater. Today Bio* **24**, 100899 (2024). 10.1016/j.mtbio.2023.100899 PMC10770530 38188644

[74] Suhail, S. et al. Engineered skin tissue equivalents for product evaluation and therapeutic applications. *Biotechnol. J* **14**, 1900022 (2019). 10.1002/biot.201900022 PMC6615970 30977574

[75] Chakraborty, J., Gupta, A. C. & Ghosh, S. A 3D bioprinted in vitro full-thickness skin aging model. *J. Mater. Chem. B* 12981–12999 (2025). 10.1039/d5tb01126d 40657796

[76] Park, W., Gao, G. & Cho, D.-W. Tissue-specific decellularized extracellular matrix bioinks for musculoskeletal tissue regeneration and modeling using 3d bioprinting technology. *Int. J. Mol. Sci.* **22**, 7837 (2021). 34360604 10.3390/ijms22157837 PMC8346156

[77] Fonseca, A. C. et al. Emulating human tissues and organs: a bioprinting perspective toward personalized medicine. *Chem. Rev.* **120**, 11093–11139 (2020). 32937071 10.1021/acs.chemrev.0c00342 PMC7645917

[78] Augustine, R. et al. 3D Bioprinted cancer models: revolutionizing personalized cancer therapy. *Transl. Oncol.* **14**, 101015 (2021). 33493799 10.1016/j.tranon.2021.101015 PMC7823217

[79] Wei, Q. et al. Three-dimensional bioprinting of tissue-engineered skin: Biomaterials, fabrication techniques, challenging difficulties, and future directions: a review. *Int. J. Biol. Macromol* **266**, 131281 (2024). 38641503 10.1016/j.ijbiomac.2024.131281

[80] Lian, L. et al. Uniaxial and coaxial vertical embedded extrusion bioprinting. Adv. *Healthc. Mater.* **11**, 2102411 (2022). 10.1002/adhm.202102411 34860472

[81] Ventisette, I. et al. Gold-hydrogel nanocomposites for high-resolution laser-based 3D printing of scaffolds with SERS-sensing properties. *ACS Appl. Bio Mater* **7**, 4497–4509 (2024). 10.1021/acsabm.4c00379 PMC11253086 38925631

[82] Liu, J. et al. Simple and robust 3D bioprinting of full-thickness human skin tissue. *Bioengineered* **13**, 10090–10100 (2022). 10.1080/21655979.2022.2063651 PMC9161989 35412953

[83] Corrò, C., Novellasdemunt, L. & Li, V. S. W. A brief history of organoids. *Am. J. Physiol. Cell Physiol.* **319**, C151–C165 (2020). 32459504 10.1152/ajpcell.00120.2020 PMC7468890

[84] Rheinwatd, J. G. & Green, H. Seria cultivation of strains of human epidemal keratinocytes: the formation keratinizin colonies from single cells. *Cell* **6**, 331–343 (1975). 1052771 10.1016/s0092-8674(75)80001-8

[85] Sun, H., Zhang, Y.-X. & Li, Y.-M. Generation of skin organoids: potential opportunities and challenges. *Front. Cell Dev. Biol.* **9**, 709824 (2021). 34805138 10.3389/fcell.2021.709824 PMC8600117

[86] Hong, Z.-X. et al. Bioengineered skin organoids: from development to applications. *Mil. Med. Res.* **10**, 40 (2023). 37605220 10.1186/s40779-023-00475-7 PMC10463602

[87] Sun, T. et al. Culture of skin cells in 3D rather than 2D improves their ability to survive exposure to cytotoxic agents. *J. Biotechnol.* **122**, 372–381 (2006). 16446003 10.1016/j.jbiotec.2005.12.021

[88] West, H. C. & Bennett, C. L. Redefining the role of Langerhans cells as immune regulators within the skin. *Front. Immunol.* **8**, 1941 (2018). 29379502 10.3389/fimmu.2017.01941 PMC5770803

[89] Roets, B. Potential application of PBM use in hair follicle organoid culture for the treatment of androgenic alopecia. *Mater. Today Bio* **23**, 100851 (2023). 10.1016/j.mtbio.2023.100851 PMC10663892 38024838

[90] Liu, Y. et al. Sebaceous gland organoid engineering. *Burns Trauma.* **12**, tkae003 (2024). 38699464 10.1093/burnst/tkae003 PMC11063650

[91] Jung, S.-Y. et al. Wnt-activating human skin organoid model of atopic dermatitis induced by Staphylococcus aureus and its protective effects by Cutibacterium acnes. *iScience* **25** (2022). 10.1016/j.isci.2022.105150 PMC9526179 36193049

[92] Zheng, F. et al. Patient-specific organoid and organ-on-a-chip: 3D cell-culture meets 3D printing and numerical simulation. *Adv. Biol.* **5**, 2000024 (2021). 10.1002/adbi.202000024 PMC8243895 33856745

[93] Lee, J. & Koehler, K. R. Skin organoids: a new human model for developmental and translational research. *Exp. Dermatol.* **30**, 613–620 (2021). 33507537 10.1111/exd.14292 PMC8265774

[94] Kim, Yena, & Ji Hyeon Ju. Generation of 3D skin organoid from cord blood-derived induced pluripotent stem cells. J. Vis. Exp. **146.103791**, 59297 (2019). 10.3791/59297 31058887

[95] Ramovs, V. et al. Characterization of the epidermal-dermal junction in hiPSC-derived skin organoids. *Stem Cell Rep.* **17**, 1279–1288 (2022). 10.1016/j.stemcr.2022.04.008 PMC9213820 35561682

[96] Kwak, S. et al. Development of pluripotent stem cell-derived epidermal organoids that generate effective extracellular vesicles in skin regeneration. *Biomaterials* **307**, 122522 (2024). 38428092 10.1016/j.biomaterials.2024.122522

[97] Boonekamp, K. E. et al. Long-term expansion and differentiation of adult murine epidermal stem cells in 3D organoid cultures. *Proc. Natl. Acad. Sci. USA* **116**, 14630–14638 (2019). 31253707 10.1073/pnas.1715272116 PMC6642409

[98] Lee, J. et al. Hair-bearing human skin generated entirely from pluripotent stem cells. *Nature* **582**, 399–404 (2020). 32494013 10.1038/s41586-020-2352-3 PMC7593871

[99] Lee, J. et al. Generation and characterization of hair-bearing skin organoids from human pluripotent stem cells. *Nat. Protoc.* **17**, 1266–1305 (2022). 35322210 10.1038/s41596-022-00681-y PMC10461778

[100] Ahmed, I. et al. An optimized protocol for generating appendage-bearing skin organoids from human-induced pluripotent stem cells. *Biol. Methods Protoc.* **9**, bpae019 (2024). 38605978 10.1093/biomethods/bpae019 PMC11009018

[101] Marinho, P. A. et al. The development of an in vitro human hair follicle organoid with a complexity similar to that in vivo. *Biomed. Mater.* **19**, 025041 (2024). 10.1088/1748-605X/ad2707 38324888

[102] Lee, J. et al. Hair follicle development in mouse pluripotent stem cell-derived skin organoids. *Cell Rep* **22**, 242–254 (2018). 29298425 10.1016/j.celrep.2017.12.007 PMC5806130

[103] Bloor, A. et al. A phase I trial of iPSC-derived MSCs (CYP-001) in steroid-resistant acute GvHD. *Blood* **132**, 4562–4562 (2018).

[104] Huang, C. Y. et al. Population-based high-throughput toxicity screen of human iPSC-derived cardiomyocytes and neurons. *Cell Rep*. **39** (2022). 10.1016/j.celrep.2022.110643 35385754

[105] Ma, J. et al. Application of an iPSC-derived organoid model for localized scleroderma therapy. *Adv. Sci.* **9**, 2106075 (2022). 10.1002/advs.202106075 PMC9165518 35315234

[106] Muller, Q. et al. Development of an innervated tissue-engineered skin with human sensory neurons and Schwann cells differentiated from iPS cells. *Acta Biomater* **82**, 93–101 (2018). 30316025 10.1016/j.actbio.2018.10.011

[107] Dubau, M. et al. Development of an iPSC-derived immunocompetent skin model for identification of skin sensitizing substances. *J. Tissue Eng.* **16**, 20417314251336296 (2025). 40336952 10.1177/20417314251336296 PMC12056326

[108] Ou, Lingling, et al. Patient-derived melanoma organoid models facilitate the assessment of immunotherapies. *EBioMedicine* **92** (2023). 10.1016/j.ebiom.2023.104614 PMC10277922 37229906

[109] Zhou, S. et al. Role of the tumor microenvironment in malignant melanoma organoids during the development and metastasis of tumors. *Front. Cell Dev. Biol.* **11**, 1166916 (2023). 37152280 10.3389/fcell.2023.1166916 PMC10154581

[110] Ma, J. et al. Establishment of human pluripotent stem cell-derived skin organoids enabled pathophysiological model of SARS-CoV-2 infection. *Adv. Sci.* **9**, 2104192 (2022). 10.1002/advs.202104192 PMC8895131 34971128

[111] Li, P. et al. Mpox virus infection and drug treatment modelled in human skin organoids. *Nat. Microbiol.* **8**, 2067–2079 (2023). 37828248 10.1038/s41564-023-01489-6

[112] Kim, H. et al. Potency classification of isothiazolinone compounds based on defined approaches of skin sensitization in OECD GL 497. Environ. *Anal. Health Toxicol.* **38**, e2023026–0 (2023). 10.5620/eaht.2023026 PMC10834078 38298045

[113] Zambuto, S. G., Scott, A. K. & Oyen, M. L. FDA modernization act 2.0 and reproductive research. *Nat. Rev. Bioeng.* **2**, 984–986 (2024).

[114] Lee, S. et al. Guidelines for manufacturing and application of organoids: skin. *Int. J. Stem Cells* **17**, 182–193 (2024). 38783680 10.15283/ijsc24045 PMC11170114

[115] Leung, C. M. et al. A guide to the organ-on-a-chip. *Nat. Rev. Methods Primers* **2**, 33 (2022).

[116] Wan, H.-Y. et al. Stabilization and improved functionality of three-dimensional perfusable microvascular networks in microfluidic devices under macromolecular crowding. *Biomater. Res.* **27**, 32 (2023). 37076899 10.1186/s40824-023-00375-w PMC10116810

[117] Lam, J. et al. A microphysiological system-based potency bioassay for the functional quality assessment of mesenchymal stromal cells targeting vasculogenesis. *Biomaterials* **290**, 121826 (2022). 36201944 10.1016/j.biomaterials.2022.121826

[118] Lee, J. S. et al. Hybrid skin chips for toxicological evaluation of chemical drugs and cosmetic compounds. *Lab Chip* **22**, 343–353 (2022). 34904990 10.1039/d1lc00550b

[119] Jusoh, N., Ko, J. & Jeon, N. L. Microfluidics-based skin irritation test using in vitro 3D angiogenesis platform. *APL Bioeng.* **3** (2019). 10.1063/1.5093975 PMC6697035 31431937

[120] Sun, S. et al. Modeling human HSV infection via a vascularized immune-competent skin-on-chip platform. *Nat. Commun.* **13**, 5481 (2022). 36123328 10.1038/s41467-022-33114-1 PMC9485166

[121] Risueño, I. et al. Skin-on-a-chip models: General overview and future perspectives. *APL Bioeng*. **5** (2021). 10.1063/5.0046376 PMC8270645 34258497

[122] Driver, R. & Mishra, S. Organ-on-a-chip technology: an in-depth review of recent advancements and future of whole body-on-chip. *BioChip J.* **17**, 1–23 (2023).

[123] Wagner, I. et al. A dynamic multi-organ-chip for long-term cultivation and substance testing proven by 3D human liver and skin tissue co-culture. *Lab Chip* **13**, 3538–3547 (2013). 23648632 10.1039/c3lc50234a

[124] Sung, J. H. & Kim, J. J. Recent advances in in vitro skin-on-a-chip models for drug testing. *Expert Opin. Drug Metab. Toxicol.* **19**, 249–267 (2023). 37379024 10.1080/17425255.2023.2227379

[125] Palasantzas, V. E. J. M. et al. iPSC-derived organ-on-a-chip models for personalized human genetics and pharmacogenomics studies. *Trends Genet* **39**, 268–284 (2023). 36746737 10.1016/j.tig.2023.01.002

[126] Mistry, K. and Alexander, M. H. Skin-on-a-chip microfluidic devices: production, verification, and uses in cosmetic toxicology. in (Pant, A. B. et al.) Skin 3-D Models and Cosmetics Toxicity, 47–82 (Springer Nature, 2023).

[127] Hu, N. et al. Advancements in microfluidics for skin cosmetic screening. *Analyst* **148**, 1653–1671 (2023). 36960759 10.1039/d2an01716d

[128] Ismayilzada, N. et al. Skin-on-a-chip technologies towards clinical translation and commercialization. *Biofabrication* **16**, 042001 (2024). 10.1088/1758-5090/ad5f55 38964314

[129] Mikimoto, D. et al. Culture insert device with perfusable microchannels enhances in vitro skin model development and barrier function assessment. *Biofabrication* **16**, 035006 (2024). 10.1088/1758-5090/ad3a15 38569494

[130] Hofmann, E. et al. Modelling the complexity of human skin in vitro. *Biomedicines* **11**, 794 (2023). 36979772 10.3390/biomedicines11030794 PMC10045055

[131] Ko, J. et al. Microfluidic high-throughput 3D cell culture. *Nat. Rev. Bioeng.* **2**, 453–469 (2024).

[132] Berthier, E., Young, E. W. K. & Beebe, D. Engineers are from PDMS-land, biologists are from polystyrenia. *Lab Chip* **12**, 1224–1237 (2012). 22318426 10.1039/c2lc20982a

[133] Whitesides, G. M. The origins and the future of microfluidics. *Nature* **442**, 368–373 (2006). 16871203 10.1038/nature05058

[134] Sackmann, E. K., Fulton, A. L. & Beebe, D. J. The present and future role of microfluidics in biomedical research. *Nature* **507**, 181–189 (2014). 24622198 10.1038/nature13118

[135] Sriram, G. et al. Full-thickness human skin-on-chip with enhanced epidermal morphogenesis and barrier function. *Mater. Today* **21**, 326–340 (2018).

[136] Lee, H. R. & Sung, J. H. Effect of culture condition on cell viability and gel contraction in a skin chip. *J. Ind. Eng. Chem* **87**, 60–67 (2020).

[137] Valencia, L. et al. A new microfluidic method enabling the generation of multi-layered tissues-on-chips using skin cells as a proof of concept. *Sci. Rep.* **11**, 13160 (2021). 34162909 10.1038/s41598-021-91875-z PMC8222336

[138] Park, S. et al. Flow-IMPACT: a pumpless, high-throughput 3D cell culture platform for investigation of synergistic angiogenic effects. *Adv. Mater. Technol.* **10**, 2401526 (2025).

[139] Rovida, C. Local lymph node assay: how testing laboratories apply OECD TG 429 for REACH purposes. *ALTEX* **28**, 117–29 (2011). 21625828 10.14573/altex.2011.2.117

[140] Han, B.-I. et al. Evaluation of skin sensitization potential of chemicals by local lymph node assay using 5-bromo-2-deoxyuridine with flow cytometry. *Regul. Toxicol. Pharmacol.* **107**, 104401 (2019). 31158384 10.1016/j.yrtph.2019.05.026

[141] Nakajima, S. et al. Langerhans cells are critical in epicutaneous sensitization with protein antigen via thymic stromal lymphopoietin receptor signaling. *J. Allergy Clin. Immunol* **129**, 1048–1055.e6 (2012). 22385635 10.1016/j.jaci.2012.01.063 PMC4600611

[142] Toebak, M. J. et al. Dendritic cells: biology of the skin. *Contact Dermat* **60**, 2–20 (2009). 10.1111/j.1600-0536.2008.01443.x 19125717

[143] Ramadan, Q. & Ting, F. C. W. In vitro micro-physiological immune-competent model of the human skin. *Lab Chip* **16**, 1899–1908 (2016). 27098052 10.1039/c6lc00229c

[144] Sosa, S. et al. Assessment of skin sensitization properties of few-layer graphene and graphene oxide through the Local Lymph Node Assay (OECD TG 442B). *NanoImpact* **29**, 100448 (2023). 36565921 10.1016/j.impact.2022.100448

[145] Misery, L. Skin, immunity and the nervous system. Br. *J. Dermatol* **137**, 843–850 (1997). 9470898

[146] Blake, K. J., Jiang, X. R. & Chiu, I. M. Neuronal regulation of immunity in the skin and lungs. *Trends Neurosci* **42**, 537–551 (2019). 31213389 10.1016/j.tins.2019.05.005 PMC6661013

[147] Zudaire, E. et al. A computational tool for quantitative analysis of vascular networks. *PLoS ONE* **6**, e27385 (2011). 22110636 10.1371/journal.pone.0027385 PMC3217985

[148] Matthews, J. M. et al. OrganoID: a versatile deep learning platform for tracking and analysis of single-organoid dynamics. *PLoS Comput. Biol.* **18**, e1010584 (2022). 36350878 10.1371/journal.pcbi.1010584 PMC9645660

[149] Choi, D.-H. et al. Analyzing angiogenesis on a chip using deep learning-based image processing. *Lab Chip* **23**, 475–484 (2023). 36688448 10.1039/d2lc00983h

[150] Kim, S. et al. Angio-Net: deep learning-based label-free detection and morphometric analysis of in vitro angiogenesis. *Lab Chip* **24**, 751–763 (2024). 38193617 10.1039/d3lc00935a

[151] Song, E. et al. VONet: A deep learning network for 3D reconstruction of organoid structures with a minimal number of confocal images. Patterns 5 2024. 10.1016/j.patter.2024.101063 PMC11573902 39569212

[152] Zhang, J. et al. Construction of a high fidelity epidermis-on-a-chip for scalable in vitro irritation evaluation. *Lab Chip* **21**, 3804–3818 (2021). 34581381 10.1039/d1lc00099c

[153] Nicolas, A. et al. High throughput transepithelial electrical resistance (TEER) measurements on perfused membrane-free epithelia. *Lab Chip* **21**, 1676–1685 (2021). 33861225 10.1039/d0lc00770f

[154] Tokuyama, M. & Mabuchi, T. New treatment addressing the pathogenesis of Psoriasis. *Int. J. Mol. Sci.* **21**, 7488 (2020). 33050592 10.3390/ijms21207488 PMC7589905

[155] Garshick, M. S. et al. Inflammasome signaling and impaired vascular health in Psoriasis. *Arterioscler. Thromb. Vasc. Biol.* **39**, 787–798 (2019). 30760013 10.1161/ATVBAHA.118.312246 PMC6436998

[156] Boguniewicz, M. & Leung, D. Y. M. Atopic dermatitis: a disease of altered skin barrier and immune dysregulation. *Immunol. Rev.* **242**, 233–246 (2011). 21682749 10.1111/j.1600-065X.2011.01027.x PMC3122139

[157] Kim, B. E. & Leung, D. Y. M. Significance of skin barrier dysfunction in Atopic Dermatitis. *Allergy Asthma Immunol. Res* **10**, 207–215 (2018). 29676067 10.4168/aair.2018.10.3.207 PMC5911439

[158] Jung, S.-y et al. Wnt-activating human skin organoid model of atopic dermatitis induced by Staphylococcus aureus and its protective effects by Cutibacterium acnes. *iScience* **25**, 105150 (2022). 36193049 10.1016/j.isci.2022.105150 PMC9526179

[159] Moldoveanu, D. et al. Spatially mapping the immune landscape of melanoma using imaging mass cytometry. *Sci. Immunol* **7**, eabi5072 (2022). 35363543 10.1126/sciimmunol.abi5072

[160] Ayuso, J. M. et al. Microfluidic model with air-walls reveals fibroblasts and keratinocytes modulate melanoma cell phenotype, migration, and metabolism. *Lab Chip* **21**, 1139–1149 (2021). 33533390 10.1039/d0lc00988a PMC7990711

[161] Roudsari, L. C. & West, J. L. Studying the influence of angiogenesis in in vitro cancer model systems. *Adv. Drug Deliv. Rev.* **97**, 250–259 (2016). 26571106 10.1016/j.addr.2015.11.004

[162] Flont, M., Dybko, A. & Jastrzębska, E. A layered cancer-on-a-chip system for anticancer drug screening and disease modeling. *Analyst* **148**, 5486–5495 (2023). 37768020 10.1039/d3an00959a

[163] Vázquez-Aristizabal, P. et al. Biofabrication and monitoring of a 3D printed skin model for melanoma. Adv. Healthc. Mater. 2401136. 10.1002/adhm.202401136 PMC12344628 38992996

[164] Schmidle, P. et al. Lives of skin lesions in monkeypox: histomorphological, immunohistochemical, and clinical correlations in a small case series. *Viruses* **15**, 1748 (2023). 37632089 10.3390/v15081748 PMC10458687

[165] Dahiya, N. et al. Hyper-parameter tuned deep learning approach for effective human monkeypox disease detection. *Sci. Rep.* **13**, 15930 (2023). 37741892 10.1038/s41598-023-43236-1 PMC10517970

[166] Zeyen, C. et al. Clinical spectrum and long-term outcomes of mpox: a cohort study spanning from acute infection to six-month follow-up. *BMC Infect. Dis.* **24**, 317 (2024). 38491447 10.1186/s12879-024-09191-6 PMC10941457

[167] Zhu, J. & Abaci, H. E. Human skin-on-a-chip for mpox pathogenesis studies and preclinical drug evaluation. *Trends Pharmacol. Sci.* **44**, 865–868 (2023). 37500295 10.1016/j.tips.2023.07.001 PMC10811284

[168] OECD (2015), Test No. 430: In Vitro Skin Corrosion: Transcutaneous Electrical Resistance Test Method (TER), OECD Guidelines for the Testing of Chemicals, Section 4, OECD Publishing, Paris, 10.1787/9789264242739-en.

[169] OECD (2025), Test No. 431: In vitro skin corrosion: reconstructed human epidermis (RHE) test method, OECD Guidelines for the Testing of Chemicals, Section 4, OECD Publishing, Paris, 10.1787/9789264264618-en.

[170] OECD (2025), Test No. 439: In Vitro Skin Irritation: Reconstructed Human Epidermis Test Method, OECD Guidelines for the Testing of Chemicals, Section 4, OECD Publishing, Paris, 10.1787/9789264242845-en.

[171] OECD (2025), Test No. 437: Bovine Corneal Opacity and Permeability Test Method for Identifying i) Chemicals Inducing Serious Eye Damage and ii) Chemicals Not Requiring Classification for Eye Irritation or Serious Eye Damage, OECD Guidelines for the Testing of Chemicals, Section 4, OECD Publishing, Paris, 10.1787/9789264203846-en.

[172] OECD (2023), Test No. 438: Isolated Chicken Eye Test Method for Identifying i) Chemicals Inducing Serious Eye Damage and ii) Chemicals Not Requiring Classification for Eye Irritation or Serious Eye Damage, OECD Guidelines for the Testing of Chemicals, Section 4, OECD Publishing, Paris, 10.1787/9789264203860-en.

[173] OECD (2025), Test No. 492: Reconstructed human Cornea-like Epithelium (RhCE) test method for identifying chemicals not requiring classification and labelling for eye irritation or serious eye damage, OECD Guidelines for the Testing of Chemicals, Section 4, OECD Publishing, Paris, 10.1787/9789264242548-en.

[174] OECD (2024), Test No. 492B: Reconstructed Human Cornea-like Epithelium (RHCE) Test Method for Eye Hazard Identification, OECD Guidelines for the Testing of Chemicals, Section 4, OECD Publishing, Paris, 10.1787/0d603916-en.

[175] Vickerman, V. et al. Design, fabrication and implementation of a novel multi-parameter control microfluidic platform for three-dimensional cell culture and real-time imaging. *Lab Chip* **8**, 1468–1477 (2008). 18818801 10.1039/b802395f PMC2560179

[176] Shin, J. et al. Monolithic digital patterning of polydimethylsiloxane with successive laser pyrolysis. *Nat. Mater.* **20**, 100–107 (2021). 32807919 10.1038/s41563-020-0769-6

[177] Fernandes Quero, R., Jesus, D. P. & Fracassi da Silva, J. A. Fracassi da Silva, Simple modification to allow high-efficiency and high-resolution multi-material 3D-printing fabrication of microfluidic devices. *Lab Chip* **23**, 3694–3703 (2023). 37477358 10.1039/d3lc00356f

[178] Lee, Y. et al. Microfluidics within a well: an injection-molded plastic array 3D culture platform. *Lab Chip* **18**, 2433–2440 (2018). 29999064 10.1039/c8lc00336j

[179] Abaci, H. E. et al. Nat. Commun. 9, 5301 (2018). 10.1038/s41467-018-07579-y PMC6294003 30546011

[180] Wufuer, M. et al. Skin-on-a-chip model simulating inflammation, edema and drug-based treatment. *Sci. Rep.* **6**, 37471 (2016). 27869150 10.1038/srep37471 PMC5116589

[181] Ko, J. et al. Human ocular angiogenesis-inspired vascular models on an injection-molded microfluidic chip. *Adv. Healthc. Mater* **8**, 1900328 (2019). 10.1002/adhm.201900328 31199057

[182] Kim, M. et al. An advanced 3D lymphatic system for assaying human cutaneous lymphangiogenesis in a microfluidic platform. *NPG Asia Mater* **16**, 7 (2024).

